# Fractional unit-root tests allowing for a fractional frequency flexible Fourier form trend: predictability of Covid-19

**DOI:** 10.1186/s13662-021-03317-9

**Published:** 2021-03-15

**Authors:** Tolga Omay, Dumitru Baleanu

**Affiliations:** 1 Department of Economics, Atilim University, 06830 Ankara, Turkey; 2 Department of Mathematics, Cankaya University, 06530 Balgat, Ankara, Turkey; 3 Institute of Space Sciences, Magurele-Bucharest, Romania

**Keywords:** C01, C02, C14, C15, C22, Structural break, Stochastic fractional difference equation, Stationarity, Covid-19 forecast

## Abstract

In this study we propose a fractional frequency flexible Fourier form fractionally integrated ADF unit-root test, which combines the fractional integration and nonlinear trend as a form of the Fourier function. We provide the asymptotics of the newly proposed test and investigate its small-sample properties. Moreover, we show the best estimators for both fractional frequency and fractional difference operator for our newly proposed test. Finally, an empirical study demonstrates that not considering the structural break and fractional integration simultaneously in the testing process may lead to misleading results about the stochastic behavior of the Covid-19 pandemic.

## Introduction

The forecasts of daily events lead many decision-making processes to be more manageable. In time-series analysis, forecasts are generally made using the Box–Jenkins method. In the Box–Jenkins method, the prerequisite for making long-term forecasting or setting up an ARIMA model is the stationarity of the series under investigation. It is also essential to make long-term forecasts in the current Covid-19 outbreak. These forecasts contain crucial information to eliminate the uncertainties that may arise during the process. For example, forecasting the peak number of infected cases in the long term may give valuable information about the health care system. If these numbers can be accurately predicted, then the intensive care unit bed capacities and other resources can be allocated efficiently. This vital information can also be used by the other sectors which are affected by the Covid-19 outbreak. Besides, long-term forecasting can also be made for all other natural phenomena. Reliable forecasts of earthquakes, meteorology, biodiversity, and others are needed to manage disasters. The time-series literature has described covariance stationarity as a steady state in which the mean, variance, and covariance do not change over time. The stochastic difference equation’s stationarity is determined by using the unit-root test of [1]. The test’s basic principle is to see if the first-degree stochastic difference equation’s parameter is statistically equal to 1 or not. If it is equal to 1, the series is a unit-root process or simply not stationary.

In this study, we need to add the dynamics of this natural outbreak to the [1] (henceforth, ADF) method to examine the outbreak’s stochastic features and test its long-term predictability. If the epidemic’s data generation process is substituted correctly into the test methodology leading to a stationarity test result, then we can claim that the correct long-term forecast model is achieved. It is recognized that the number of daily cases in the outbreak models conforms to exponential function patterns. However, it is not easy to generalize the epidemic model to different functional designs, such as the second wave that may occur in later stages. This functional pattern will create a double exponential model or a more complex functional form. The complexities that arise obtained in this way can also decrease the effectiveness of the long-term forecasts. We have used the Fourier function to overcome this problem, thereby providing a remarkable convergence to any functional form whose structure is uncertain. In the literature, many researchers have employed the Fourier function to capture smooth structural breaks with integer frequency. Nevertheless, some studies have shown that this should be handled within a fractional frequency structure. In addition to the importance of using low frequency, previous studies have also emphasized the problems of using cumulative frequency in Fourier type of unit-root testing. A well-known problem associated with the traditional unit-root tests is that the power of the test decreases if too many variables are added into the testing equation when the cumulative frequency is employed.

So how can the Fourier function capture the short-term oscillations in the daily cases without using cumulative frequency? In the consecutive days of pandemics, different dynamics or numbers of infected patients are detected. Temporary or permanent jumps are a prevailing dynamic of daily infected cases that the first-order difference equation cannot capture. The fractional difference equation employed recently in the literature is seen to solve such dynamics. It has been observed that the number of daily cases exhibits fractional-order difference equation features. After detrending the daily infected cases data with the Fourier function, the remaining series exhibit the features of a fractional first-order difference equation. Therefore, in the light of these explanations, the pretest of the long-term predictability of the number of daily Covid-19 cases must have considered the fractional frequency Fourier functional form with a fractional difference equation. Let us now turn to discussing the methodology used in the paper and literature available until now.

Following the influential work of [1], testing the stationarity characteristics of variables has attracted a great deal of attention among researchers. This testing methodology can be broadly classified into three categories; linear unit-root tests, unit-root tests that permit a break in mean and/or trend (this can be termed time-dependent nonlinearity, or structural break (SB)), and finally unit-root tests that permit state-dependent nonlinearity. However, after recognizing the long-memory features of the stochastic processes, the fractionally integrated unit-root tests have attracted a great deal of attention in the recent literature. Therefore, in this study, we will focus on combining the unit-root tests that permit structural break and fractional integration (FI).

A typical exercise in most time-series investigations is to check whether the drift part of a series is correctly characterized as deterministic or stochastic. Naturally, the stochastic drift is considered as a unit-root process. In contrast, the deterministic one is particularly time trends. It is generally concluded that traditional methods developed for fractionally integrated processes could drive spurious FI response if employed to short memory processes encompassing structural breaks. The reverse outcome is also entirely recognized; standard methods for identifying and measuring break dates lead to spurious structural change, generally at the midpoint of the series, in fact, there is only fractional integration in the sample (see [2, 3]). Therefore, fractional integration and structural break indicate vastly diverse long and medium-run dynamics, making it hard to discriminate among them. As it is also recommended in [4], these two methods, FI and SB, are alternative methods for difference stationarity (D-ST) and trend stationarity (T-ST). It is well known that to avoid spurious estimates of the parameters and biases in the time-series studies, the data must be differenced to make them stationary. Therefore, the decision of optimal differencing is vital for obtaining correct information from the data under investigation. By introducing these two alternatives, we have to choose the correct differencing among D-ST, T-ST, fractional difference stationarity (FD-ST), and structural break stationarity (SB-ST). In addition to these, it is well documented that in the D-ST case memory is infinite, and past shocks are perfectly remembered. In the case of T-ST, memory is short, and the autocorrelation function decays exponentially. [2] indicates that the FI processes or the FD-ST case establish an interesting alternative to this separation as they are capable of linking the gap between these two possibilities. Therefore, FD-ST has a long memory but not as much as the D-ST, which indicates that $d = 0.1$ has a short memory with respect to $d = 0.9$. These methods can fulfil the gap between the short-lasting and unchanging effect of shocks in the T-ST and D-ST models, respectively, by providing transitional behaviors such as long memory and nonstationary mean-reversion (see [2]). So finding the exact differencing order is vital to limit information losses. As we have mentioned above, fractional integration and structural break indicate very different medium and long-run dynamics. Thus, it is hard to differentiate between them, so it is essential first to distinguish these two methodologies. To this end, we propose a procedure that combines these two methods using a simple but yet efficient way to identify the SB and FI processes correctly. Therefore, we can eliminate the problems which are explained in the above paragraph efficiently.

The unit-root tests which are permitting for a break in mean and/or trend are as follows; [5–8], and [9]. These have acknowledged alternative trend models in examining for the unit-root testing, and have concentrated on models with segmented line trends; and single or multiple breaks [10]. However, recent studies have proposed unit-root tests where the alternative hypothesis is stationarity around a smoothly changing trend. [11] (LNV, hereafter) and [12] used logistic smooth trend functions that permit a smooth break in the data’s deterministic trend. [13] specified nonlinear trend employing Chebyshev polynomials. Reference [14] employed trigonometric functions in Fourier form to define probable smooth breaks in the data. Numerous problems were encountered with these types of unit-root tests.^1^ Nevertheless, the simplest and most accurate one has been the Fourier function, which was used by [14–16], and [17].

The second strand of literature deals with the fractionally integrated unit-root test proposed by [18] (henceforth, DGM). One stated that that both null hypotheses were rejected frequently in the previous studies, and concluded that many time-series were not well characterized as either $I ( 1 )$ or $I ( 0 )$. Therefore, the group of fractionally integrated processes, represented as $FI ( d )$, has proved to be very suitable in catching the persistence features of many long-memory processes (see [19, 20], and [21]). Reference [18] has pointed out the shortcomings of the alternative methodologies used and suggested a simple Wald-type test in the time domain with adequate power properties. As a by-product of its application, this test delivers knowledge about the values of *d* under the alternative hypothesis. Therefore, this methodology is a generalization of the well-known Dickey–Fuller (D-F) test, which was originally developed for the case of $I ( 1 )$ versus $I ( 0 )$, to the more general case of $FI ( d_{0} )$ versus $FI ( d_{1} )$ with $d_{1} < d_{0}$ and, thus, is denoted as the fractional Dickey–Fuller (FD-F) test. DGM test is based on the normalized-OLS estimates, or on its *t*-ratio, of the parameter on $\Delta ^{d_{1}}y_{t - 1}$ in a regression of $\Delta ^{d_{0}}y_{t}$ on $\Delta ^{d_{1}}y_{t - 1}$ and possibly some lags of $\Delta ^{d_{0}}y_{t}$. Depending on the alternative hypothesis $H_{1}:d < d_{0}$, the pre-estimation is needed for the order of *d*. DGM has shown that the choice of a $T^{1/2}$ consistent estimator of *d* in its appropriate range suffices to make the FD-F test possible, while preserving asymptotic normality. Reference [18] has highlighted the advantages of their testing procedure as follows. The first one is theorizing the simple D-F framework to obtain simplicity for testing unit roots with a fractional difference operator. The second one is that the LM tests proposed contain a different structure than the traditional LM tests. The proposed LM test does not assume any known density for errors, which makes it more robust to fundamental ones. The third one is that in the exact case where $d_{0} = 1$, the FD-F method inherits the flexibility of the standard D-F test. This provides a usual framework for testing the $I(1)$ null hypothesis against some interesting compound alternative. According to [18], producing a fractional integration unit-root test by including a structural break does not seem feasible with other FI unit-root tests. However, the flexible FD-F structure that they propose will make this study much easier and feasible. The final one is that [18] has found a good finite sample properties with respect to other competing tests.

Following [18], the third advice, we have extended this methodology to the structural break set up by using the [17] method. As we have mentioned above, the [17] procedure employs trigonometric functions in the form of Fourier form to define presumable smooth breaks in the data. Numerous difficulties are encountered with structural break type of unit-root tests. Nevertheless, the easiest and accurate one is the Fourier function used by [17] with which extended it to fractional frequency case. Therefore, [17] is another simple generalization of the ADF test like the DGM test. Combining these two simple methodologies will emerge as a more generalized and simple set up without facing any unnecessary details to test stationarity in a composite alternative hypothesis. The composite hypothesis of the series under investigation is a fractionally integrated series around a smoothly changing trend.

Other attempts have been made in the literature to combine these two methodologies (namely SB and FI) by using different techniques. References [22] and [23], following [24] and [25], derived a Lagrange multiplier test in the time domain, and [26] and [3] have considered Wald-type tests for a unit-root null hypothesis against fractional integration following [18]. The traditional unit-root tests usually reject the null hypothesis when the actual process is a series that is integrated fractionally with $d= (0.5,1)$. We will see later that such series are not stationary. Therefore, the results of these studies become questionable. Moreover, it is well known that short memory processes with level shifts display features that lead one to conclude that long memory is present in the data generating process (e.g., [23], among many others). On the other hand, it was also recognized that long-memory processes cause the null hypothesis of no structural change to be rejected when traditional structural change tests are used (see, [2, 3, 23], among many others). To overcome these problems in the SB-FI literature and to address the reasons mentioned earlier, we propose the SB-FI unit-root test in the form of a fractionally integrated series around a smoothly changing fractional frequency flexible Fourier form. Therefore, we have obtained the following contributions from this newly proposed methodology:

1. The confusion about structural break and fractional integration, which we explained above, has been resolved with the most appropriate methods.
2. The two-step methodology allowed us to obtain the asymptotic distribution of the unit-root test easily.
3. It has been shown that the Fourier function can represent the deterministic structure of the Covid-19 outbreak. Also the best optimization algorithm that should be used with the fractional frequency Fourier function is found.
4. For fractional integration, a new estimator has been proposed that minimizes information losses. It has also been shown that predictions can be made with the least loss of information with this new estimator.
5. Finally, how to design the optimal forecast model for outbreaks by combining all of these methodologies has been shown.

The structure of the article is as follows. Section 2 presents fractional frequency Fourier form fractionally integrated ADF test with its asymptotic distribution and presents an extensive simulation study to show the small-sample features. Section 3 discusses the various optimization algorithms that can be used with the fractional frequency estimation along with the parametric and semi parametric estimation of the difference operator *d*. Section 4 applies the FFFFF-FI-ADF test to pretest the long-term predictability of the Covid-19 cases. Section 5 is devoted to concluding remarks.

## The methodology for the fractional frequency flexible Fourier form fractionally integrated ADF test: FFFFF-FI-ADF

In the introduction, we gave some basic ideas about the testing procedure. The main concern is to be simple in deriving the test, and its asymptotic. Hence, we have started with the Fourier approach in which we can detrend the series at first and assume the remaining part has fractionally integrated stationarity or nonstationarity of the series. Apart from [15, 16] and [17], this two-step approach provides a straightforward setting for obtaining the testing procedure and asymptotic distribution of the proposed test statistics. Therefore, we will start with the Fourier approach and include the fractionally integrated ADF test in the second step.

References [16] and [17] consider the following augmented Dickey–Fuller (DF) test:

1 $$ y_{t} = \varphi (t) + \psi y_{t - 1} + \lambda t + \varepsilon _{t} , $$

where $\varepsilon _{t}$ is a stationary error term with a variance of $\sigma ^{2}$, and $\varphi (t)$ denotes the deterministic intercept and trend. Reference [16] claims that it is problematic to estimate Eq. (1) directly and study the unit-root hypothesis $\psi = 1$ without knowing the functional structure of $\varphi (t)$. Following [14, 16, 27] and [17], we assume that $\varphi (t)$ includes the following Fourier components:

2 $$ \varphi (t) = \alpha _{0} + \alpha _{1}\sin \biggl( \frac{2\pi kt}{T} \biggr) + \alpha _{2}\cos \biggl( \frac{2\pi kt}{T} \biggr), $$

where $\alpha _{0}$, $\alpha _{1}$, and $\alpha _{2}$ are changing intercept parameters, *T* is the number of observations, and *t* gives the trend term. The term *k* denotes the particular frequency to be determined over a pre-given interval. The trigonometric components $\sin ( \frac{2\pi kt}{T} )$ and $\sin ( \frac{2\pi kt}{T} )$ are utilized to approximate smooth breaks. If $\alpha _{1} = \alpha _{2} = 0$, then there are no smooth breaks. Through the grid-search method, [15, 16] use $k = k^{*}$ to minimize the residual sum of squares (SSR) in Eq. (1), where $k^{*}$ indicates the value of *k* that achieves the minimum SSR. Besides, Becker et al. (2006) show that it is acceptable to set $k=1$ or $k=2$ to find the substantial structural changes in the data. Using a data-driven technique, [14] set the maximum number of breaks to be 5. Reference [15] further recommends the usage of low frequency to capture the smooth structural changes in the data. Reference [17] mentions the flexibility of the integer, but argues that it has many drawbacks in estimating the smooth trends (i.e., over filtration, type two error etc.). Hence, we follow [17] and use the fractional version of the test in this paper. To this end, instead of searching for a single integer frequency *k* in Eq. (2) we try to find the fractional frequency in Eq. (3), which is also employed in [14] and [15, 16] for integer values. The largest frequency applied is $k_{\max } $, and $\Delta k = 0.1$ is used in the 0.1 range and other smaller increments, and the accuracy of the fractional frequency search was increased. The optimal fractional frequency is obtained at the point where the SSR is the lowest. This optimization process is carried out by applying the algorithm described above for Eq. (1). Moreover, we can also employ this to define the fractional frequency Fourier trend by using an *F*-test as proposed in [14] and [15, 16]. The model is as follows:

3 $$ \Delta y_{t} = \alpha _{0} + \alpha _{1}\sin \biggl( \frac{2\pi kt}{T} \biggr) + \alpha _{2}\cos \biggl( \frac{2\pi kt}{T} \biggr) + \delta y_{t - 1} + \lambda t + \varepsilon _{t} . $$

The null hypothesis of linear unit root is obtained when $\delta = 0$, which is suggested by [16]. The two-step testing process is as follows.

In the first step of the two stages procedure the following regression is run:

4 $$ y_{t} = \alpha _{0} + \alpha _{1}\sin \biggl( \frac{2\pi k^{fr}t}{T} \biggr) + \alpha _{2}\cos \biggl( \frac{2\pi k^{fr}t}{T} \biggr) + \lambda t + \bar{\omega }_{t}, \quad t = 1,2,\ldots,T , $$

where $k^{fr}$ indicates the fractional Fourier frequency. The above equation assumes that $\omega _{t}$ is a random walk process and after being demeaned or detrended it can be used in the second step as $\bar{\omega }_{t}$,

5 $$ \bar{\omega }_{t} = \delta \bar{\omega }_{t - 1} + u_{t}, $$

where $u_{t} \sim iidN ( 0,\sigma _{u}^{2} )$ and the initial condition $\bar{\omega }_{0}$ is a constant. Notice that this technique is asymptotically the same as the one step procedure of [17].

As we have mentioned above instead of assuming the case of $I ( 1 )$ versus $I ( 0 )$, the more general case of $FI ( d_{0} )$ versus $FI ( d_{1} )$ with $d_{1} < d_{0}$ can be used following [18]. The DGM test is based on the normalized-OLS estimates, or on its *t*-ratio, of the coefficient on $\Delta ^{d_{1}}\bar{\omega }_{t - 1}$ in a regression of $\Delta ^{d_{0}}\bar{\omega }_{t}$ on $\Delta ^{d_{1}}\bar{\omega }_{t - 1}$ and possibly some lags of $\Delta ^{d_{0}}\bar{\omega }_{t}$.^2^ The definition of the $FI(d)$ process that we will implement is that of an (asymptotically) stationary process when $d < 0.5$, and that of a nonstationary (truncated) process when $d > 0.5$.

For the asymptotic distribution of $\delta = 1$, the two-step process will be used with the following demeaned and detrended series *ω̄*:

$$\begin{aligned}& \bar{\omega }_{t} = y_{t - 1} - \hat{\alpha }_{0} - \hat{\alpha }_{1}\sin \biggl( \frac{2\pi \hat{k}^{fr}t}{T} \biggr) - \hat{\alpha }_{2}\cos \biggl( \frac{2\pi \hat{k}^{fr}t}{T} \biggr), \\& \bar{\omega }_{t} = y_{t - 1} - \hat{\alpha }_{0} - \hat{\alpha }_{1}\sin \biggl( \frac{2\pi \hat{k}^{fr}t}{T} \biggr) - \hat{\alpha }_{1}\cos \biggl( \frac{2\pi \hat{k}^{fr}t}{T} \biggr) - \hat{\lambda } t, \end{aligned}$$

where $\hat{\alpha }_{0}$, $\hat{\alpha }_{1}$, $\hat{\alpha }_{2}$ and *λ̂* are OLS estimators for demeaned and detrended cases, respectively. Next, we build the fractional Fourier unit-root test by using the demeaned and detrended series $\bar{\omega }_{t}$ in the second step. Although the D-F test is coherent when compared to the fractional alternatives, its low power makes it an appropriate ground for studying the new test procedures. Thus, we extend the regression model in (3) and (5) to test the null hypothesis that a series is $FI(d_{0})$ against the alternative that it is $FI(d_{1})$. The variable *ω̄* is thought to be a unit-root process under the null hypothesis, but it constitutes a fractionally integrated stationary process in the alternative. Precisely, our suggestion is built upon testing for the statistical significance of *β* in the following FI-DF equation:

6 $$ \Delta ^{d_{0}}\bar{\omega }_{t} = \beta \Delta ^{d_{1}} \bar{\omega }_{t - 1} + \xi _{t} , $$

where $\xi _{t} \sim iid ( 0,\sigma _{\xi }^{2} )$ $I(0)$ process. Keep in mind that (6) is still an unbalanced regression where the dependent and independent variables are differenced with respect to their degrees of integration under the null and the alternative hypothesis. The $\bar{\omega }_{t}$ series follows the following process assuming that $u_{t} = \xi _{t}$ and $\beta = 0$ in (6):

7 $$ \Delta ^{d_{0}}\bar{\omega }_{t} = \xi _{t}. $$

This implies that $\bar{\omega }_{t}$ in (7) is $FI(d_{0})$. When $\beta < 0$, $\bar{\omega }_{t}$ can be expressed as

8 $$ \bigl( \Delta ^{d_{0} - d_{1}} - \beta L \bigr)\Delta ^{d_{1}}\bar{\omega }_{t} = \xi _{t} , $$

where $\bar{\omega }_{t}$ is a $FI(d_{1})$ process. By using these arguments, we can write the normalized-OLS estimated coefficient or its *t*-ratio as in the standard D-F testing methodology as follows:

$$ \textstyle\begin{array}{ll} H_{0}:\beta = 0,\bar{\omega }_{t}\text{ is }FI(d_{0}) \quad\quad & \bullet \text{ Linear unit root }I(1),\\ H_{1}:\beta < 0,\bar{\omega }_{t} \text{ is }FI(d_{1})\quad\quad & \bullet \text{ Fractionally integrated around a smoothly} \\ &\hphantom{\bullet } \text{ changing trend} \varphi (t) - FI(d_{1}) . \end{array} $$

### The test and its asymptotic properties

Now we allow for $d_{0} = 1$ and $u_{t} = \xi _{t}$ in (7), where $\{ \xi _{t} \} $ is a sequence of zero-mean i.i.d. random variables with unknown variance $\sigma _{\xi }^{2}$ and finite fourth-order moment. The OLS estimator $\hat{\beta }_{ols}$ and its *t*-ratio, $t_{FF}$, are given by their usual least-squares formulas;

9 $$\begin{aligned} & \hat{\beta }_{ols} = \frac{\sum_{t = 2}^{T} \Delta \bar{\omega }_{t}\Delta ^{d_{1}}\bar{\omega }_{t - 1}}{\sum_{t = 2}^{T} ( \Delta ^{d_{1}}\bar{\omega }_{t - 1} )^{2}}, \end{aligned}$$

10 $$\begin{aligned} & t_{FF} = \frac{\sum_{t = 2}^{T} \Delta \bar{\omega }_{t}\Delta ^{d_{1}}\bar{\omega }_{t - 1}}{S_{T} ( \sum_{t = 2}^{T} ( \Delta ^{d_{1}}\bar{\omega }_{t - 1} )^{2} )^{1/2}}, \\ & S_{T}^{2} = \frac{\sum ( \Delta \bar{\omega }_{t} - \hat{\beta }_{ols}\Delta ^{d_{1}}\bar{\omega }_{t - 1} )^{2}}{T}. \end{aligned}$$

In order to obtain the asymptotic distribution of the $t_{FF} ( i = \mu ,\tau )$ test, we need the subsequent outcomes, where we let $[ rT ]$, $r \in [ 0,1 ]$ be an integer close to *rT*. During the course of the derivation → implies weak convergence as *T* approaches ∞.

#### Proposition 1



We have assumed that the remaining part or detrended series is a fractionally integrated series. Thus, we preserve the notation of [18] hereafter to derive the asymptotics of the proposed test.

#### Lemma 1

*Let* $\{ \xi _{t} \} $ *be a sequence of zero*-*mean i*.*i*.*d*. *random variables with variance* $\sigma _{\xi }^{2}$ *such that* $E \vert \xi _{t}^{4} \vert < \infty $ *implies the following linear processes*:

$$\begin{aligned}& \Delta ^{d}z_{t} = \xi _{t} , \quad d \in [ - 0.5,0.5 ),\\& \Delta ^{d}z_{t}^{*} = \xi _{t}I_{t > 0}, \quad d \in [ - 0.5,0.5 ), \end{aligned}$$

*where* $I(\cdot )$ *is an indicator function and*

$$ g_{t}^{*} = \sum_{i = 1}^{t} z_{i}^{*}. $$

*Then the following process verifies*:

Case 1 $$\begin{aligned}& \textit{if } - 0.5 < d < 0.5, \\& T^{ - 1}\sum_{t = 1}^{T} \bigl( z_{t} - z_{t}^{*} \bigr) = o_{p}(1), \end{aligned}$$

11 $$\begin{aligned}& T^{ - 1}\sum_{t = 1}^{T} \bigl( z_{t}^{2} - z_{t}^{*^{2}} \bigr) = o_{p}(1), \end{aligned}$$

*and*

12 $$\begin{aligned}& T^{ - 1}\sum_{t = 1}^{T} \bigl( z_{t}z_{t + k} - z_{t}^{*}z_{t + k}^{*} \bigr) = o_{p}(1), \\& \textit{if } d = - 0.5 \\& (T\log T)^{ - 1}\sum_{t = 1}^{T} z_{t}^{*^{2}} \overset{p}{\rightarrow}\frac{\sigma ^{2}}{\pi }; \end{aligned}$$

*where* $\overset{p}{\rightarrow}$ *denotes convergence in probability*,

Case 1 $$\begin{aligned}& \textit{if } - 0.5 < d < 0.5 , \\& T^{ - 2 ( 1 + d )}\sum_{t = 1}^{T} z_{t}^{*^{2}} \overset{w}{\rightarrow} \int _{0}^{1} W_{d}^{2}\,dr, \end{aligned}$$

*where* $\overset{p}{\rightarrow}$ *denotes weak convergence*.

#### Lemma 2

*Let* $\xi _{t}$, $z_{t}$, $z_{t}^{*}$, *and* $g_{t}^{*}$ *be identified as in Lemma *1. *Then the subsequent processes are martingale differences and confirm*:

* $$\begin{aligned}& \textit{if } 0 < d < 0.5, \\& T^{ - 1/2}\sum_{t = 2}^{T} z_{t - 1}\xi _{t}\overset{w}{\rightarrow} N \biggl( 0,\sigma ^{4}\frac{\Gamma ( 1 - 2d )}{\Gamma ^{2} ( 1 - d )} \biggr), \end{aligned}$$

13 $$\begin{aligned}& T^{ - 1/2}\sum_{t = 2}^{T} z_{t - 1}^{*}\xi _{t}\overset{w}{\rightarrow}N \biggl( 0,\sigma ^{4}\frac{\Gamma ( 1 - 2d )}{\Gamma ^{2} ( 1 - d )} \biggr), \end{aligned}$$

Case 2 $$\begin{aligned}& \textit{if } d = - 0.5 \\& (T\log T)^{ - 1}\sum_{t = 1}^{T} z_{t}^{*}\xi _{t} \overset{w}{\rightarrow}N \biggl( 0,\frac{\sigma ^{4}}{\pi } \biggr); \\& \textit{if } - 0.5 < d < 0. \end{aligned}$$

When we impose the Fourier form to the FI process:

13 $$\begin{aligned}& T^{ - ( 1 + d )}\sum_{t = 2}^{T} g_{t - 1}\xi _{t}\overset{w}{\rightarrow} \sigma ^{2} \int _{0}^{1} B_{d} \bigl( k^{fr},r \bigr)\,dB ( r ), \end{aligned}$$

14 $$\begin{aligned}& T^{ - ( 1 + d )}\sum_{t = 2}^{T} g_{t - 1}^{*}\xi _{t}\overset{w}{\rightarrow}\sigma ^{2} \int _{0}^{1} W_{d} \bigl( k^{fr},r \bigr)\,dB ( r ), \end{aligned}$$

where $B(\cdot )$ denotes a standard Brownian motion, and $B_{d}(\cdot )$, $W_{d}(\cdot )$ or $W_{d}(k^{fr},r)$ are standard fractional Brownian motions. Depending on Lemma 2, the subsequent two theorems derive the asymptotic distribution and prove the consistency of an appropriately standardized-OLS estimator of *β̂* and its *t*-ratio, under the null hypothesis of $I ( 1 )$.

#### Theorem 1

*Under the null hypothesis of unit root and with* $\bar{\omega }_{t}$ *as a random walk*, *the asymptotic distribution of* $t_{FF}$ *is as follows*: *where* $W_{i, - d_{1}} ( k^{fr},r )$ *for* $i = \mu ,\tau $ *give the demeaned and detrended standard fractional Brownian motions*.

We can derive the asymptotics of the other cases; $d_{1} = 0.5$ and $0.5 < d_{1} < 1$ in a similar fashion with the $0 \le d_{1} < 0.5$. As pointed out before, since we are following two-step approaches the other distributions are the same as [18]. Therefore, we concentrate on the non-degenerated distribution of case 1 and give its distribution explicitly in Theorem 1. This asymptotic distribution obtained for fractional frequency is the general form of the integer frequency case, and it can be easily converted to an integer form with the values given in [15].

#### Proof

The proof of Theorem 1 is given explicitly in Appendix A. □

Apparently, the asymptotic distribution of the obtained test statistics under the null depends on the fractional Fourier frequency, $k^{fr}$, and integration order, $d_{1}$, but it is invariant to the other parameters in the testing equation. The fractional frequency versions of the critical values are tabulated in Appendix B and for integer frequency the critical values tabulated in Tables 1–3 as follows:

**Table 1:** Critical values for FI models only intercept included in integer frequency Fourier function

*T*	100			250			500		
	1%	5%	10%	1%	5%	10%	1%	5%	10%
*k* = 1									
0.1	−4.297	−3.635	−3.295	−4.210	−3.565	−3.227	−4.175	−3.580	−3.262
0.2	−4.035	−3.377	−3.026	−3.858	−3.295	−2.970	−3.969	−3.330	−3.001
0.3	−3.777	−3.099	−2.762	−3.625	−3.011	−2.672	−3.675	−3.009	−2.676
0.4	−3.506	−2.868	−2.510	−3.386	−2.732	−2.398	−3.444	−2.780	−2.419
0.5	−3.275	−2.604	−2.260	−3.155	−2.506	−2.142	−3.212	−2.500	−2.159
0.6	−3.060	−2.406	−2.048	−2.851	−2.233	−1.898	−2.995	−2.290	−1.941
0.7	−2.860	−2.217	−1.875	−2.790	−2.077	−1.727	−2.812	−2.102	−1.745
0.8	−2.777	−2.094	−1.729	−2.706	−1.999	−1.618	−2.613	−1.935	−1.600
0.9	−2.667	−1.963	−1.604	−2.522	−1.839	−1.499	−2.583	−1.870	−1.512
*k* = 2									
0.1	−3.804	−3.172	−2.809	−3.851	−3.163	−2.804	−3.809	−3.119	−2.781
0.2	−3.737	−3.015	−2.655	−3.632	−2.963	−2.625	−3.628	−2.968	−2.629
0.3	−3.571	−2.854	−2.470	−3.431	−2.771	−2.428	−3.431	−2.755	−2.415
0.4	−3.305	−2.656	−2.292	−3.229	−2.579	−2.232	−3.271	−2.613	−2.239
0.5	−3.170	−2.473	−2.111	−3.029	−2.406	−2.051	−3.124	−2.399	−2.032
0.6	−3.058	−2.326	−1.953	−2.912	−2.170	−1.819	−2.882	−2.192	−1.838
0.7	−2.944	−2.182	−1.818	−2.759	−2.068	−1.700	−2.809	−2.069	−1.695
0.8	−2.648	−1.995	−1.661	−2.594	−1.902	−1.554	−2.594	−1.890	−1.540
0.9	−2.586	−1.926	−1.554	−2.480	−1.825	−1.461	−2.523	−1.837	−1.483
*k* = 3									
0.1	−3.636	−2.964	−2.616	−3.614	−2.989	−2.657	−3.510	−2.941	−2.589
0.2	−3.505	−2.847	−2.514	−3.492	−2.830	−2.485	−3.420	−2.810	−2.491
0.3	−3.311	−2.698	−2.344	−3.364	−2.668	−2.323	−3.304	−2.657	−2.315
0.4	−3.246	−2.549	−2.213	−3.222	−2.509	−2.157	−3.191	−2.485	−2.142
0.5	−3.072	−2.430	−2.050	−2.968	−2.326	−1.974	−3.044	−2.306	−1.959
0.6	−2.944	−2.269	−1.878	−2.819	−2.171	−1.817	−2.857	−2.205	−1.848
0.7	−2.867	−2.123	−1.761	−2.755	−2.054	−1.666	−2.737	−2.012	−1.628
0.8	−2.775	−2.006	−1.661	−2.644	−1.937	−1.546	−2.611	−1.941	−1.578
0.9	−2.577	−1.905	−1.554	−2.480	−1.827	−1.474	−2.498	−1.807	−1.453
*k* = 4									
0.1	−3.579	−2.926	−2.563	−3.526	−2.909	−2.564	−3.563	−2.880	−2.561
0.2	−3.466	−2.758	−2.425	−3.399	−2.758	−2.435	−3.415	−2.756	−2.402
0.3	−3.355	−2.670	−2.283	−3.246	−2.588	−2.248	−3.233	−2.623	−2.281
0.4	−3.206	−2.528	−2.144	−3.087	−2.408	−2.065	−3.185	−2.441	−2.108
0.5	−3.099	−2.347	−2.009	−2.962	−2.294	−1.945	−2.994	−2.331	−1.942
0.6	−3.011	−2.270	−1.899	−2.821	−2.130	−1.786	−2.820	−2.136	−1.788
0.7	−2.829	−2.094	−1.724	−2.706	−2.030	−1.672	−2.680	−1.988	−1.623
0.8	−2.670	−1.994	−1.628	−2.510	−1.919	−1.558	−2.617	−1.888	−1.525
0.9	−2.634	−1.897	−1.536	−2.488	−1.821	−1.439	−2.552	−1.892	−1.492
*k* = 5									
0.1	−3.555	−2.861	−2.525	−3.445	−2.866	−2.540	−3.484	−2.801	−2.494
0.2	−3.431	−2.724	−2.391	−3.383	−2.726	−2.368	−3.345	−2.694	−2.374
0.3	−3.333	−2.578	−2.246	−3.269	−2.568	−2.241	−3.290	−2.576	−2.234
0.4	−3.136	−2.465	−2.131	−3.034	−2.420	−2.088	−3.058	−2.413	−2.064
0.5	−2.975	−2.334	−1.969	−2.955	−2.299	−1.932	−2.945	−2.291	−1.930
0.6	−2.961	−2.187	−1.810	−2.797	−2.095	−1.742	−2.838	−2.140	−1.801
0.7	−2.808	−2.092	−1.708	−2.713	−2.001	−1.642	−2.655	−1.987	−1.642
0.8	−2.684	−2.009	−1.640	−2.609	−1.917	−1.546	−2.599	−1.909	−1.550
0.9	−2.650	−1.909	−1.537	−2.571	−1.874	−1.484	−2.509	−1.813	−1.444

**Table 2:** Critical values for FI models intercept and trend included in integer Fourier function

*T*	100			250			500		
	1%	5%	10%	1%	5%	10%	1%	5%	10%
*k* = 1									
0.1	−4.722	−4.110	−3.809	−4.649	−4.054	−3.765	−4.567	−4.003	−3.708
0.2	−4.505	−3.865	−3.511	−4.320	−3.722	−3.447	−4.257	−3.698	−3.397
0.3	−4.183	−3.524	−3.199	−4.089	−3.409	−3.087	−4.031	−3.394	−3.073
0.4	−3.931	−3.205	−2.883	−3.730	−3.107	−2.781	−3.723	−3.035	−2.717
0.5	−3.640	−2.951	−2.606	−3.515	−2.837	−2.484	−3.414	−2.732	−2.392
0.6	−3.367	−2.752	−2.382	−3.220	−2.536	−2.174	−3.127	−2.444	−2.079
0.7	−3.167	−2.502	−2.167	−3.044	−2.345	−1.987	−2.806	−2.202	−1.882
0.8	−2.985	−2.282	−1.933	−2.843	−2.143	−1.777	−2.733	−2.020	−1.671
0.9	−2.857	−2.170	−1.782	−2.720	−2.007	−1.620	−2.557	−1.916	−1.530
*k* = 2									
0.1	−4.524	−3.869	−3.519	−4.402	−3.775	−3.458	−4.369	−3.764	−3.453
0.2	−4.253	−3.606	−3.261	−4.130	−3.541	−3.197	−4.099	−3.501	−3.178
0.3	−4.122	−3.343	−3.006	−3.887	−3.273	−2.941	−3.883	−3.223	−2.878
0.4	−3.764	−3.147	−2.766	−3.599	−2.955	−2.633	−3.570	−2.924	−2.591
0.5	−3.548	−2.868	−2.510	−3.360	−2.712	−2.342	−3.349	−2.636	−2.288
0.6	−3.353	−2.689	−2.316	−3.159	−2.453	−2.122	−3.063	−2.410	−2.056
0.7	−3.051	−2.422	−2.062	−2.903	−2.233	−1.900	−2.905	−2.192	−1.818
0.8	−2.969	−2.234	−1.884	−2.753	−2.116	−1.730	−2.685	−2.005	−1.643
0.9	−2.755	−2.084	−1.724	−2.593	−1.961	−1.596	−2.598	−1.866	−1.524
*k* = 3									
0.1	−4.318	−3.649	−3.310	−4.209	−3.602	−3.273	−4.189	−3.561	−3.258
0.2	−4.055	−3.445	−3.093	−4.031	−3.392	−3.053	−3.919	−3.337	−2.998
0.3	−3.899	−3.231	−2.885	−3.822	−3.115	−2.791	−3.771	−3.097	−2.768
0.4	−3.650	−3.026	−2.680	−3.476	−2.870	−2.527	−3.504	−2.832	−2.491
0.5	−3.478	−2.791	−2.446	−3.324	−2.677	−2.327	−3.262	−2.588	−2.246
0.6	−3.311	−2.569	−2.212	−3.112	−2.473	−2.092	−3.019	−2.344	−1.978
0.7	−3.084	−2.407	−2.038	−2.843	−2.209	−1.858	−2.824	−2.177	−1.821
0.8	−2.947	−2.240	−1.884	−2.811	−2.084	−1.727	−2.656	−1.986	−1.636
0.9	−2.863	−2.142	−1.763	−2.660	−1.977	−1.608	−2.543	−1.870	−1.496
*k* = 4									
0.1	−4.157	−3.508	−3.167	−4.141	−3.487	−3.166	−4.145	−3.495	−3.146
0.2	−4.025	−3.344	−2.962	−3.912	−3.269	−2.946	−3.911	−3.240	−2.932
0.3	−3.903	−3.178	−2.816	−3.778	−3.088	−2.754	−3.704	−3.013	−2.692
0.4	−3.727	−2.941	−2.604	−3.519	−2.858	−2.505	−3.408	−2.819	−2.479
0.5	−3.411	−2.761	−2.392	−3.290	−2.639	−2.286	−3.160	−2.543	−2.203
0.6	−3.201	−2.525	−2.173	−3.151	−2.399	−2.053	−2.996	−2.328	−1.970
0.7	−3.031	−2.368	−1.998	−2.847	−2.211	−1.879	−2.837	−2.126	−1.762
0.8	−2.856	−2.209	−1.854	−2.714	−2.078	−1.724	−2.691	−2.002	−1.639
0.9	−2.853	−2.113	−1.741	−2.708	−1.973	−1.610	−2.497	−1.863	−1.513
*k* = 5									
0.1	−4.212	−3.454	−3.111	−4.021	−3.411	−3.113	−4.052	−3.405	−3.077
0.2	−3.994	−3.302	−2.939	−3.874	−3.231	−2.903	−3.787	−3.202	−2.894
0.3	−3.816	−3.114	−2.758	−3.626	−3.006	−2.663	−3.602	−2.988	−2.672
0.4	−3.522	−2.895	−2.552	−3.429	−2.783	−2.466	−3.427	−2.740	−2.430
0.5	−3.458	−2.683	−2.318	−3.267	−2.605	−2.260	−3.173	−2.532	−2.188
0.6	−3.270	−2.544	−2.177	−3.012	−2.368	−2.027	−3.032	−2.342	−1.965
0.7	−3.100	−2.390	−2.001	−2.872	−2.231	−1.896	−2.840	−2.121	−1.735
0.8	−2.887	−2.241	−1.869	−2.801	−2.093	−1.726	−2.727	−1.991	−1.612
0.9	−2.734	−2.101	−1.745	−2.693	−1.968	−1.617	−2.596	−1.865	−1.490

**Table 3:** Estimated frequencies with different optimization algorithms

	Linear trend	Grid search	Simplex	Genetic	BHHH	BFGS
Case1 $k^{fr} = 0.25$, $\alpha _{1} = 5$, $\alpha _{2} = 3$						
Est. Fre.	–	0.230	0.263	0.263	1.277	4.026
SSR	18.585	0.000	0.001	0.001	19.402	33.907
Case2 $k^{fr} = 0.25$, $\alpha _{1} = 11$, $\alpha _{2} = - 5$						
Est. Fre.	–	0.230	0.230	0.230	0.230	0.569
SSR	15.325	0.000	0.000	0.000	0.000	36.329
Case 3 $k^{fr} = 0.80$, $\alpha _{1} = - 11$, $\alpha _{2} = 1$						
Est. Fre.	–	0.690	0.689	0.689	0.689	0.687
SSR	1209.888	0.008	0.000	0.000	0.000	0.006
Case 4 $k^{fr} = 1$, $\alpha _{1} = 11$, $\alpha _{2} = - 1$						
Est. Fre.	–	0.920	0.918	0.918	0.918	0.927
SSR	1209.888	0.008	0.000	0.000	0.000	0.006
Case 5 $k^{fr} = 1.25$, $\alpha _{1} = 10$, $\alpha _{2} = - 1$						
Est. Fre.	–	1.150	1.148	1.148	1.148	1.132
SSR	4907.469	0.052	0.000	0.000	0.000	2.507

*Note:* Estimated frequency: Est. Fre. SSR: Sum of square residuals.

### Small sample properties of the fractional frequency flexible Fourier form fractionally integrated ADF test FFFFFFI-ADF (FFFFF-FI-ADF)

**Figure 1:**
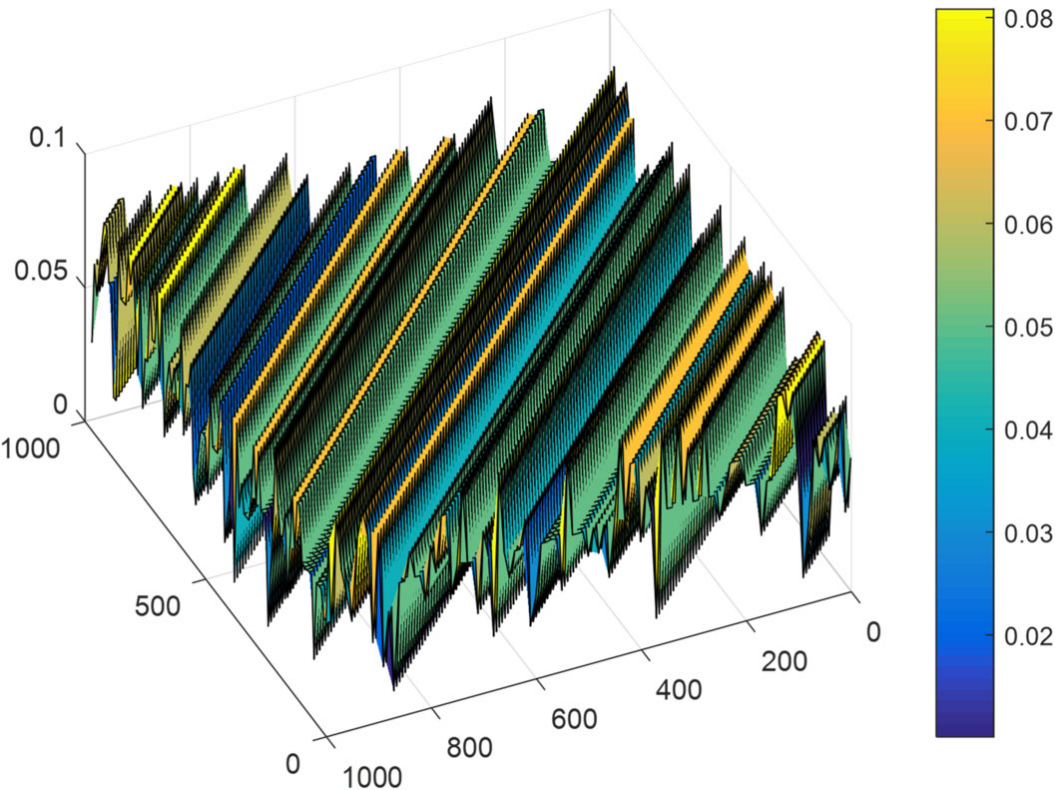
Size properties of FFFFF-FI-ADF % 5 nominal significance level

First, we will examine the small-sample size features of the test statistics. To assess the size of the test statistics, we investigate the following data generating process (DGP):

15 $$ y_{t} = y_{t - 1} + \upsilon _{t} \quad \text{for } t = 1,2,\ldots,T, \upsilon _{t} \sim iid.N ( 0,1 ) , $$

where $\upsilon _{t}$ is stationary with the above given distribution. The size features of the tests were simulated with 2000 replications via the sample dimension $T = \{ 100,200,300,400,500, 600,700,800,900,1000 \} $. The results of these simulation exercises are presented below in Fig. 1 and suggest that the proposed test statistics have satisfactory size properties.

As can be seen from Fig. 1, the newly proposed test exhibits good size properties similar to the previous tests [18, 26, 28]. Considering the scale next to the figure, the minimum and maximum size values are in the range of 0.02 and 0.08, respectively. Since the size analysis is performed for the 5 percent significance level, this scale indicates that the newly proposed test approaches the correct size value with a minimal error rate. As can be seen from the color spectrum given above, instead of the extreme values of yellow and dark blue, the size results were obtained with light green and blue intensity, and the real value of size was mostly 5%. Thus, in the light of Fig. 1 we can safely conclude that the newly proposed test has strong size properties.

Therefore, we can proceed with the power analysis without any size adjustment. Now, we turn to the small-sample power properties of the proposed tests. We have done an extensive simulation study to see the proposed unit-root tests’ power surface using the model

16 $$\begin{aligned} & y_{t} = \varphi ( t ) + \bar{\omega }_{t} , \end{aligned}$$

17 $$\begin{aligned} & \varphi (t) = \alpha _{0} + \alpha _{1}\sin \biggl( \frac{2\pi kt}{T} \biggr) + \alpha _{2}\cos \biggl( \frac{2\pi kt}{T} \biggr) , \end{aligned}$$

18 $$\begin{aligned} & y_{t} = \alpha _{0} + \alpha _{1}\sin \biggl( \frac{2\pi k^{fr}t}{T} \biggr) + \alpha _{2}\cos \biggl( \frac{2\pi k^{fr}t}{T} \biggr) + \lambda t + \bar{\omega }_{t}, \quad t = 1,2,\ldots,T, \end{aligned}$$

19 $$\begin{aligned} & \Delta ^{d_{0}}\bar{\omega }_{t} = \beta \Delta ^{d_{1}} \bar{\omega }_{t - 1} + \xi _{t}. \end{aligned}$$

**Figure 2:**
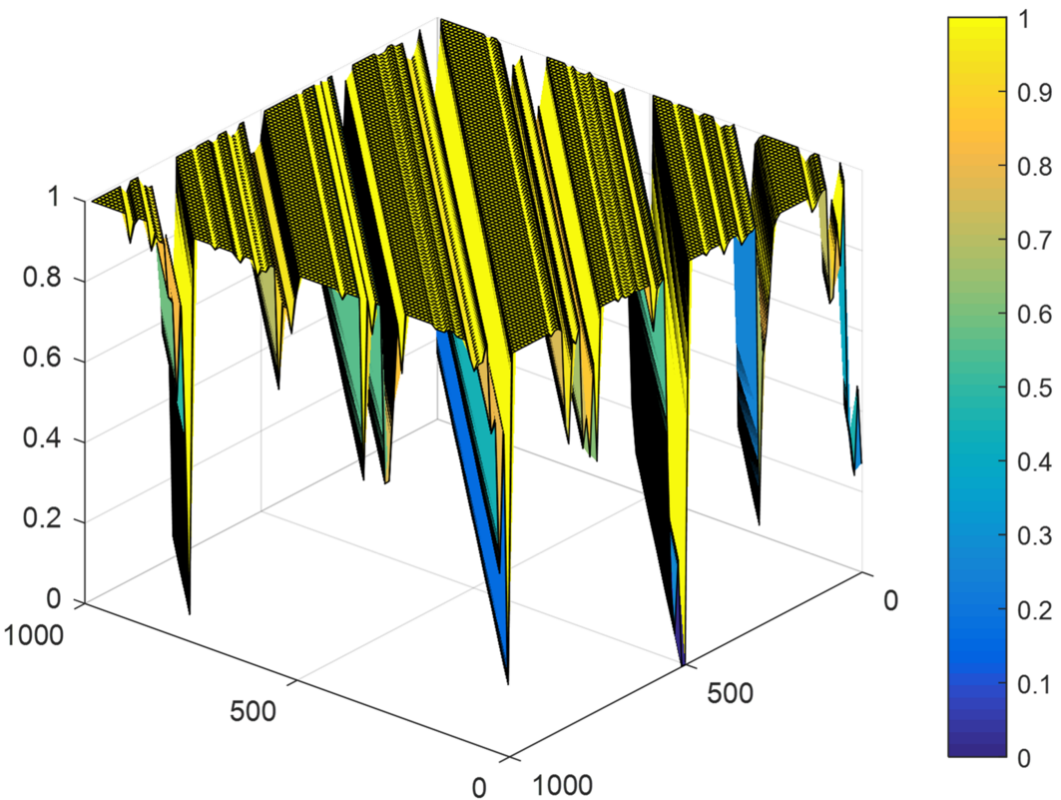
Power properties of FFFFF-FI-ADF %5 nominal significance level

Following [15, 16], we set $\alpha _{1} = ( 0,3 )$ and $\alpha _{2} = ( 0,5 )$. The results presented in Fig. 2 suggest that the proposed FFFFF-FI-ADF test clearly exhibits a similar behavior to [18, 26, 28].

As it can be seen from Fig. 2, the power performance of the test is working well. The test’s power increases with the time dimension *T* and decreases as the difference operator parameter varies from 0.1 to 0.9. Especially after 0.8, the power has started to decline from 1.00 to 0.2. and the lowest as 0.0. Towards 1, the color spectrum turns to yellow and black, while power weakens with blue and dark blue tones. As Fig. 2 shows, the high power of the test is justified with the abundance of yellow or black areas. Overall, this analysis proves that the test is powerful in capturing fractional integration data dynamics with a structural break. Furthermore, with these power analysis results we can distinguish between structural break and fractional integration because the detrended series in the first stage will not give a pseudo-integration order in the second stage.

## The method of estimations for Fourier fractional frequency and fractional integration parameter *d*

### Estimation of fractional frequency for Fourier function

**Figure 3:**
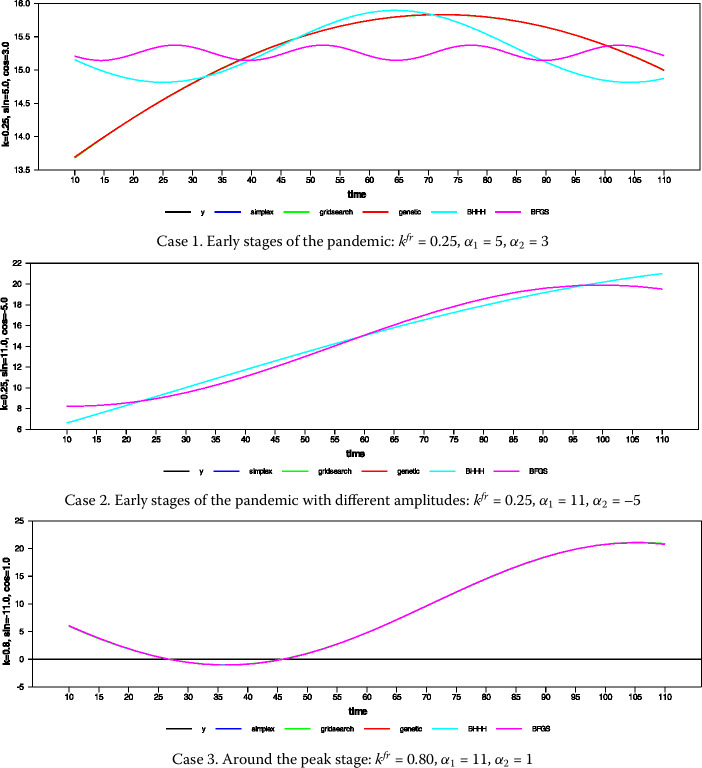

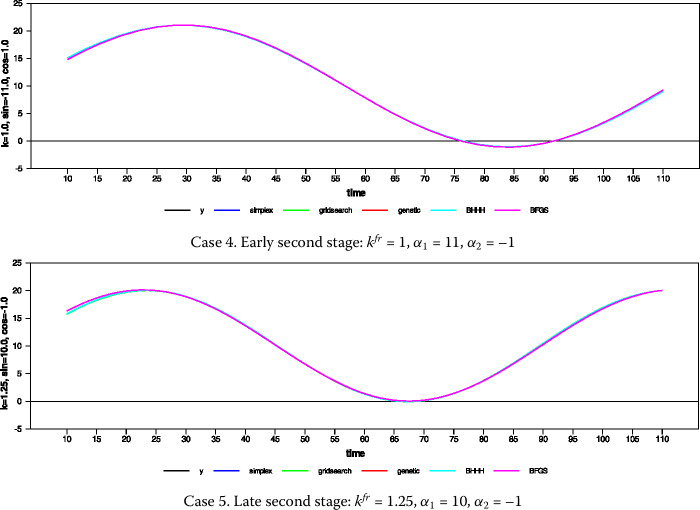
Different DGS and algorithms for obtaining fractional frequency

The authors of [29] have conducted an extensive study analyzing the BFGS, BHHH, Genetic, Simplex and Grid Search (GS) algorithms in the estimation of the fractional frequency. They used the alternative hypothesis of the test [17] to evaluate the effects of using different algorithms on the parameter estimates. They have noticed that in the earlier studies, comparison of different optimization algorithm evaluation is commonly made on the critical value accuracy. Yet the Fourier unit-root test depends on the fractional frequency. Thus, the frequency is specified at first and then the critical values are acquired. Consequently, producing the critical values with a different optimization algorithm will not lead to different set of critical values. In our simulation study, the issues in [29] will be taken into consideration. In addition, since the subject to be examined should imitate the data generation process of the Covid-19 pandemic, the following model will be used:

20 $$ y_{t} = \alpha _{0} + \alpha _{1}\sin \biggl( \frac{2\pi k^{fr}t}{T} \biggr) + \alpha _{2}\cos \biggl( \frac{2\pi k^{fr}t}{T} \biggr) , $$

where $T = 100$. Subsequently, investigating different schemes of experiments, the authors of [29] have decided to use the SSR of the estimation results. The authors of [29] have classified the fractional frequency values that they obtain in terms of the stages of the pandemic. According to this classification, the fractional frequency was estimated to be between 0–0.75 in the early stages of the pandemic, 0.75–1.0 near the peak day, 1–1.25 in the second stage, and 1.5 around the plateau stage. We follow their study and use Eq. (25) to obtain Fig. 3 and Table 3.

Like [29], we have found that the best estimation algorithms with nonlinear trends are simplex and genetic, which are indifferent in terms of SSR. As reported in [29], the second best approach appears to be the GS grid-search algorithm, while the third one is the derivative free methods of BHHH and BFGS. Consequently, following our results and the ones obtained in [29], we use the simplex algorithm for the estimation of the fractional frequency.

### Estimation of the fractional difference *d*

In this study, we have used Andrews and [30] (henceforth, AG), [24]) (henceforth, RE) and [31]) (henceforth, GPH). The authors of [31] suggest a bias-reduced log-periodogram regression estimator, $\hat{d}_{r}$, of the long-memory parameter, *d*, that eliminates the first- and higher-order biases of the GPH estimator of [31]. The bias-reduced estimator is identical to the GPH estimator except that the pseudo-regression model that produces the GPH estimator contains as extra regressors the frequencies to the power 2*k* for $k = 1,\ldots,r$ where *r* is a particular positive integer. The bias decrease is acquired by the assumptions made on the spectrum solitary in the neighborhood of the zero frequency. The authors of [30] following [24] found that the asymptotic bias, variance, and mean-squared error (MSE) of $\hat{d}_{r}$. These outcomes show that the bias of $\hat{d}_{r}$ goes to zero at a faster rate than that of the GPH. Therefore, the most suitable estimator for our FFFFF-FI-ADF test among these estimators is AG, which manages to catch the $T^{1/2}$ convergence and satisfies the unbiasedness property. There are other estimators which may be used in our study such as the [32]’s simple search algorithm. This algorithm depends on the SSR minimization and considers both the structural break and the estimation of *d*. However, since we are using the two-step procedure which considers the structural break and integration order separately, this procedure creates problems in our study. Despite this fact, we have tried the SSR approach in obtaining an estimate for the *d* parameter but found poor results with respect to the other estimators.^3^

In the light of all these results, we propose a new estimator by using a simple search algorithm, which may be more suitable in our case and many other cases. In the interval $d = [0,1]$ plenty of different dynamics are available including $d = 0$, which corresponds to stationarity, $0 < d < 0.5$, which gives difference stationarity, $0.5 \le d < 1.0$, which refers to a nonstationary but mean-reverting process, and $d = 1$, which corresponds to a unit-root process. In our case, instead of using a priori estimate of *d*, we estimate it simultaneously within the unit-root testing procedure. For this purpose, we utilize both a search algorithm and a simple bootstrap algorithm as follows.

- *Step 1:* Estimate $y_{t} = \alpha _{0} + \varphi _{1} \sin ( \frac{2\pi {kt}}{{T}} ) + \varphi _{1}\cos ( \frac{2\pi {kt}}{{T}} ) + \bar{\omega} _{{t}}$ for the series under investigation by using the optimal $k_{fr}^{*}$ and use the series thereby obtained in the second step estimation,
- *Step 2:* For a predetermined value of $d_{1}$, starting from $d_{1} = 0.1$, estimate the FFFFF-FI-ADF test value by running $\Delta ^{d_{0}}\bar{\omega }_{t} = \beta \Delta ^{d_{1}}\bar{\omega }_{t t - 1} + u_{t}$. Also introduce lags of the dependent variable using the AIC or SIC,
- *Step 3:* Obtain critical values for this predetermined value of $d_{1}$ using 2000 centered residuals from step 2 and a simple bootstrap algorithm,
- *Step 4:* Use steps 2 and 3 to obtain the *p*-values of the test statistics for the series under consideration,
- *Step 5:* Repeat steps 2 to 4 using the interval $d = (0,1)$ and increments $\Delta d = 0.001$. Then obtain all available *p*-values in this range,
- *Step 6:* If collected *p*-values truncate the 0.1 significance level, then the first truncation will be the estimate, $\hat{d}_{1}$. If there is no such truncation, then select the minimum *p*-value for the estimated $\hat{d}_{1}$ parameter.

**Figure 4:**
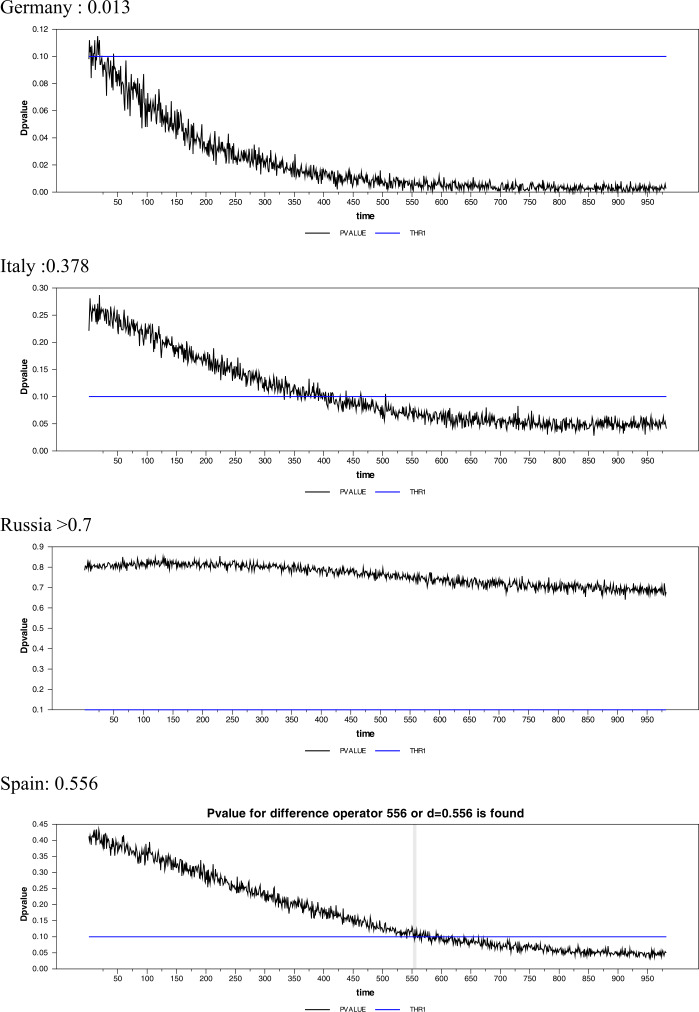

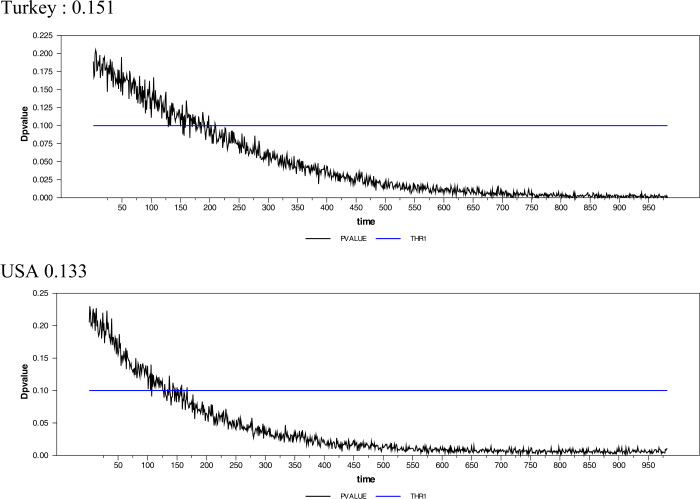
Graphical representation of the new difference estimator for unit-root test

As an example, we have obtained the estimates for Germany, Italy, Russia, Spain, Turkey, and US in Fig. 4.

Therefore, using our new methodology, we can also provide the best procedure which leads to the minimum information loss.

Let us now elaborate more on the information loss. One major drawback of differencing is that it leads to information loss. In the most extreme case, by taking the first-order difference, that is, with $d = 1$ we lose valuable information contained in a series. Taking differences is in some ways analogous to differentiation. Before taking the first-order derivative of the function we have information on its time path or the primitive function. By taking the first-order derivative of the series with respect to time, we gain information about the rate of change or growth of the series (or derived function) while passing this time path. If the subject we want to examine includes the information of the time path, a first-order derivative with respect to time will enable us to examine the series’ growth relationship.

**Figure 5:**
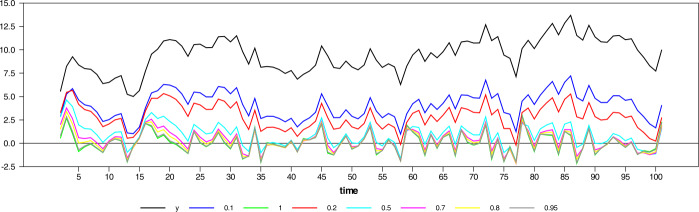
Stationary $\operatorname{AR}(1)$ process and different differencing order up to first difference

In this sense, a researcher who wants to use the gross national product (GNP) of a country must consider its growth rate because GNP in levels is not stationary. As another example, suppose we want to forecast the temperature, but the temperature data is not stationary. In order to make long-term forecasts, the series analyzed should be stationary. Otherwise, the forecast error will grow so rapidly after the one step ahead forecast that it will not allow the long-term forecast to be possible. From a forecasting perspective, it may not be relevant for the researcher to predict the growth rate of GNP instead of its level. When we take the difference from a lower order, valuable data including the growth and time path of the series is retrieved. If the *d* parameter is close to 0, the series that we obtain contains more information about the time path of the series; otherwise (for $d=1$) it conveys the growth rate of the series. On the other hand, when the difference is taken at order $d = 0.5$, an optimal mix of these two will be obtained. Figure 5 shows how the primitive function converges to the derived function as the order of differencing changes. It is obtained using the following data generation:

$$ y_{t} = 10 + 0.8y_{t - 1} + u_{t}, \quad u_{t} \sim iidN ( 0,1 ), y_{0} = 10. $$

**Figure 6:**
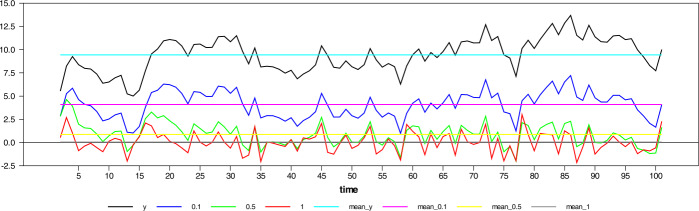
Stationary $\operatorname{AR}(1)$ process and different differencing order up to first difference with their mean values

Figure 6 visualizes the isomorphism among series with difference orders as the difference operator converges to 1.

While $d = 0.1$, the series still preserves almost all features of the original series or the time path; that is, it preserves the information about the original state of the series (primitive function) at the maximum level. However, when $d = 0.5$, the resultant series seem to resemble the series’ rate of change, although still preserving some time path information. This information has important implications in the time-series econometrics literature. Suppose the series is stationary in the interval $d = 0.1-0.5$. In that case, we can continue our work with the level of the series, i.e., time path, and obtain unbiased estimates with respect to this level information. Moreover, the traditional distribution theory is still valid while conducting regression analysis with this group of data or differenced series. But if $d> 0.5$, the series obtained no longer contains information about its time path, and we will have to comment on the rate of change. After this point, while the traditional asymptotic theory ceases to maintain its validity, we should also be careful about the different integration orders.^4^

## Empirical example

**Figure 7:**
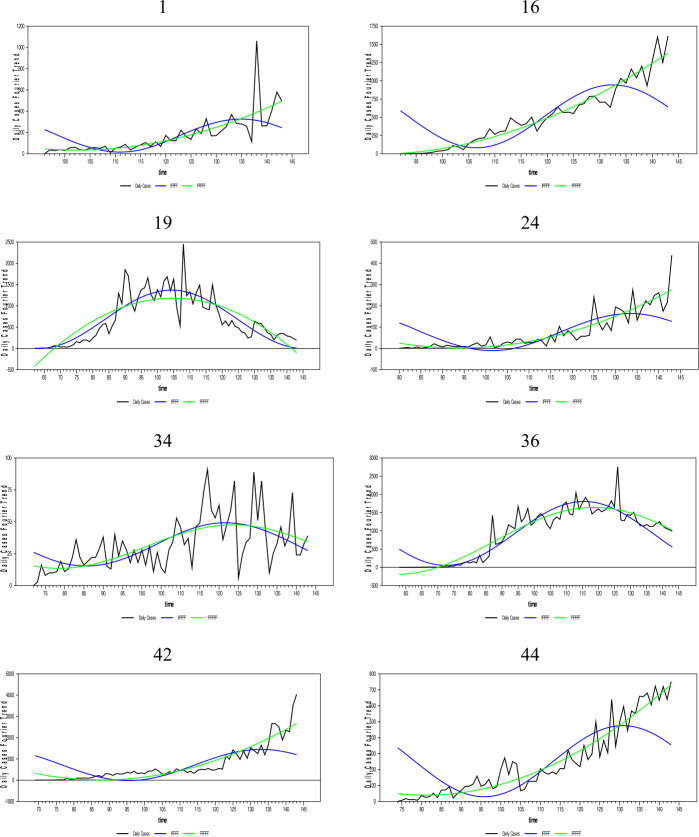

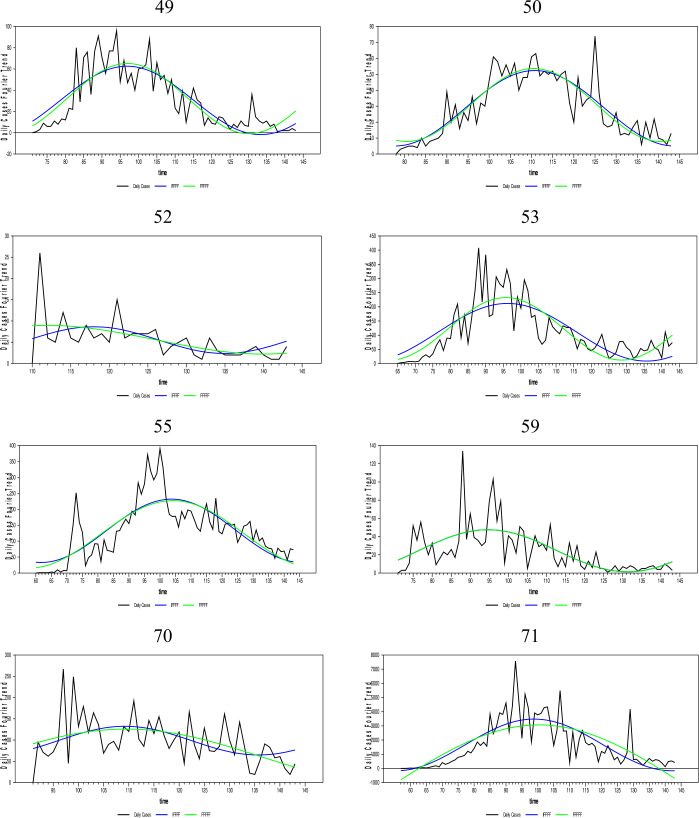

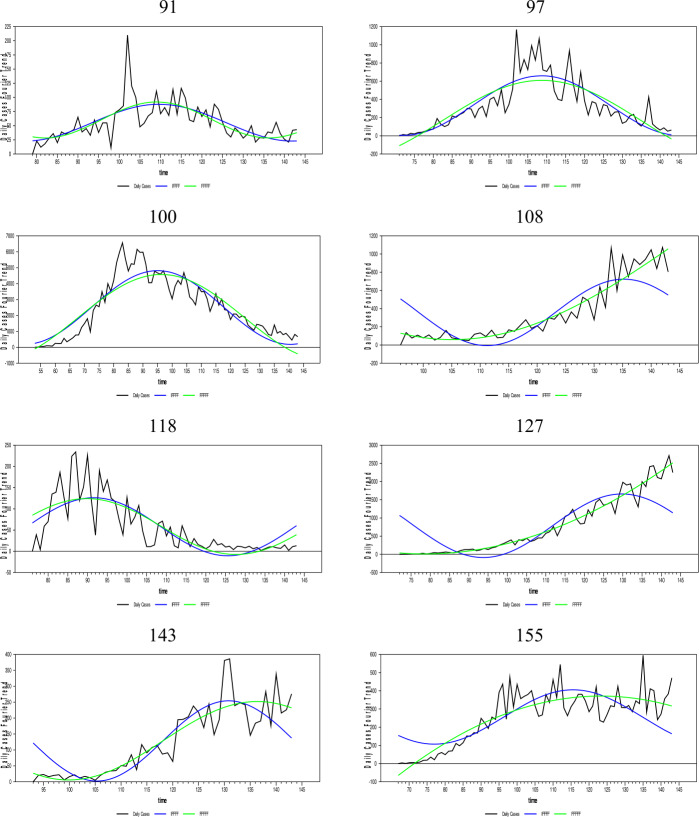

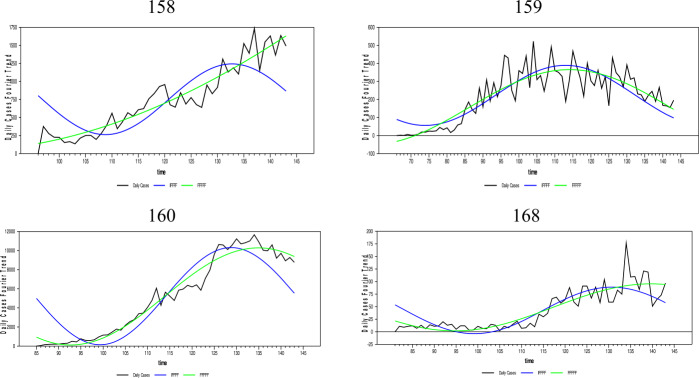
Daily infected cases and the nonlinear trends of FFFFF and IFFFF. The numbers are presenting the selected countries in alphabetic order

In this section, the daily infected case forecasts of the Coronavirus (Covid-19) pandemic, which started as of 01/01/2020 and spread worldwide, will be performed. Since the Covid-19 epidemic is on the agenda, many empirical and theoretical studies were conducted on the subject. Empirical studies on the subject include in the literature [33–37], and [38]. In addition, studies close to the theoretical structure of this article are [39–47], and [48]. The Coronavirus daily infected case numbers are collected from the European Health Organization database for 204 countries. The newly proposed FFFFF-FI-ADF type of unit-root test and the one developed in [17] were applied to the existing data of these countries. We have investigated the fit of fractional and integer Fourier functions to the daily infected case series for some selected countries by using the SSR estimates and graphed them below in Fig. 7.^5^ The countries with a longer time span that exhibit different dynamics were selected.

As Fig. 7 shows, the FFFFF method gives better results than the IFFFF method for all selected countries using the SSR criterion. Thus, these results are not tabulated. It is clear from Fig. 7 and the SSR results that the FFFFF method captures better the dynamics of the daily infected cases due to its highly flexible structure. Therefore, the FFFFF methodology can be used in obtaining the long-run forecasts of the daily Covid-19 cases. Of course, to confirm this claim, it is necessary to look at the results of both tests, namely FFFFF-ADF and FFFFF-FI-ADF.

As aforementioned, long-term forecasting is only possible with stationary data. Thus, the FFFFF methods must be used when pretesting the stationarity of the daily infected cases. Moreover, since the daily cases were found stationary using both the FFFFF-ADF and the newly proposed FFFFF-FI-ADF type of unit-root tests, a forecast model constructed for these daily cases must also include the FFFFF type of flexible function using fractional integration.

**Figure 8:**
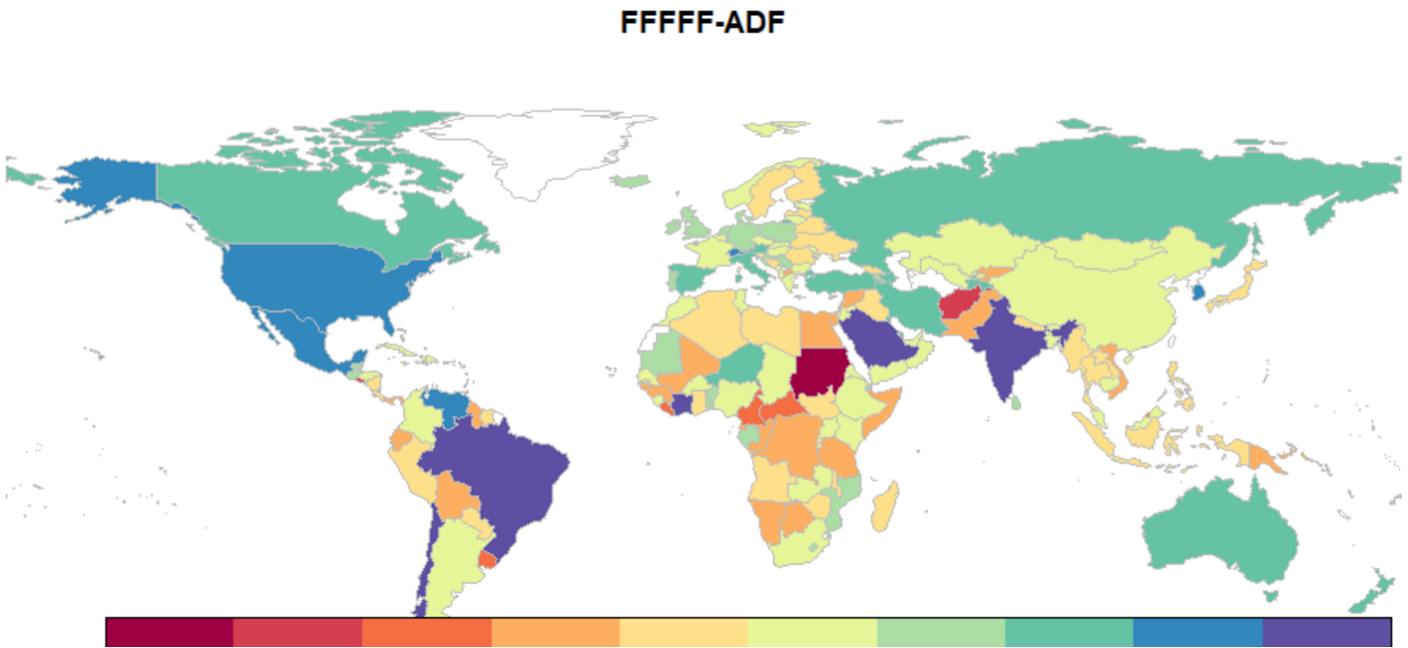
FFFFF-ADF test results with significance level tabulated on the World map

Since it would take a lot of space to tabulate the unit-root test results for the entire dataset of 240 countries, we preferred to visualize them using a world map in Fig. 8. Countries with nonstationary and stationary daily cases were colored with blue and red tones, respectively. According to the FFFFF-ADF test, the daily infected cases of 124 (out of 240) countries were found to be stationary. When we estimated the fractional frequency of the Fourier functions for these countries, 91 countries’ fractional frequencies were found in the interval 1.7 and 4.13. The high frequencies found in these countries can be attributed to the random oscillations caused by irregular testing, wrong protection measures adopted, and similar situations arising in these countries. In some countries extremely high case numbers are seen in one day, whereas the next day no tests are run, and no numbers are announced. This behavior of the health authorities leads to the irregular distribution of jump discontinuities. Despite these irregular oscillations, the fractional frequency Fourier function captures the unknown deterministic functional forms extremely well. Besides, fractional integration is also useful for capturing these random oscillations. Therefore, it is better to use the FFFFF-FI-ADF test in countries where the unit-root null could not be rejected. For this reason, we selected the ten countries with the highest daily case numbers that were not found stationary with the FFFFF-ADF test.

As can be seen from Table 4, the FFFFF-FI-ADF test results demonstrate that the daily cases of all countries, except Russia and Spain, are fractionally integrated and stationary. When it comes to Russia and Spain, their Covid-19 cases are found to be fractionally integrated, mean-reverting but nonstationary. The AG method produced stationary test results for Brazil, France, Germany, and the UK. The GPH method, which is the closest method to the AG method, yielded similar results except for Germany and the UK. Besides, the RP method leads to stationarity test results for Brazil, Chile, France, and the UK. On the other hand, the newly proposed method seems to be the most efficient one when compared to these other methods. It rejects the null hypothesis of unit root for Brazil, Chile, France, Germany, Italy, Turkey, the UK, and the US. The fractional integration dynamics that the FFFFF-ADF could not represent were caught with different methods. The oscillations that we mentioned in the introduction part was modeled correctly with FI. In this sense, it will be beneficial to use the FFFFF-FI model to forecast Covid-19’s long-term potentially infected number of cases. These efficient long-term forecasts will enable policy authorities to control the outbreak better. Moreover, we can also see that the method we have just proposed provided the lowest difference order estimates. This obtained lowest difference order allows us to perform the most accurate unit-root test with the lowest information loss.

**Table 4:** The results of the FFFFF-FI-ADF (FFFFF-FI-ADF)

FFFFF-FI-ADF	Brazil	Chile	France	Germany	Italy
AG	*d* = 0.402	*d* = 0.633	*d* = 0.256	*d* = 0.405	*d* = 0.539
	−6.688 (0.000)	−5.343 (0.000)	−5.617 (0.000)	−3.313 (0.007)	−2.257 (0.054)
New method.	*d* = 0.401	*d* = 0.006	*d* = 0.170	*d* = 0.013	*d* = 0.378
	−6.695 (0.000)	−3.709 (0.000)	−4.520 (0.000)	−3.382 (0.007)	−2.453 (0.034)
GPH	*d* = 0.393	*d* = 0.621	*d* = 0.321	*d* = 0.522	*d*>1.000
	−6.713 (0.000)	−5.350 (0.000)	−5.671 (0.000)	−3.252 (0.003)	
RE	*d* = 0.185	*d* = 0.325	*d* = 0.296	*d* = 0.583	*d* = 0.766
	−7.225 (0.000)	−5.404 (0.000)	−5.652 (0.000)	−3.213 (0.003)	−1.986 (0.045)
Frac. Fre.	0.4	0.1	1.1	1.1	1.0

FFFFF-FI-ADF	Russia	Spain	Turkey	UK	US
AG	*d* = 0.183	*d* = 0.654	*d* = 0.904	*d* = 0.147	*d*>1.000
	−1.299 (0.679)	−1.903 (0.065)	−3.122 (0.001)	−4.614 (0.003)	
New method.	*d*>0.7	*d* = 0.556	*d* = 0.151	*d* = 0.139	*d* = 0.133
		−1.955 (0.083)	−2.893 (0.065)	−5.339 (0.000)	−3.208 (0.057)
GPH	*d* = 0.289	*d* = 0.684	*d* = 0.922	*d*>1.000	*d*>1.000
	−0.972 (0.687)	−1.888 (0.064)	−3.129 (0.002)		
RE	*d* = 0.876	*d* = 0.753	*d* = 0.817	*d* = 0.317	*d* = 0.608
	0.232 (0.690)	−1.856 (0.054)	−3.087 (0.003)	−5.549 (0.000)	−3.076 (0.006)
Frac. Fre.	0.8	1.1	1.3	0.1	1.0

*Note:* Andrews and Guggenberger (2003) (AG), Robinson (1994) (RE) and Geweke and Porter-Hudak (1983) (GPH). For fractional frequency, we have used the simplex methodology. The values in the parentheses are *p*-values for related test.

## Conclusion

In this study, we have proposed a fractional frequency flexible Fourier form fractionally integrated ADF test. By implementing an extensive simulation study, we have showed that the newly proposed test has good size and power properties. Moreover, we have demonstrated that the best estimators for our unit-root testing procedure are both fractional frequency and newly proposed fractional difference operator. The newly proposed fractional difference estimator has shown to be the best estimator with respect to the minimum information loss criteria. Finally, the empirical study has demonstrated that not considering the structural break and fractional integration simultaneously in the testing process may lead to misleading results about the stochastic behavior of the series under investigation. Therefore, our proposed FFFFF-FI-ADF test will help policy authorities to control any natural disaster by providing an efficient method for pretesting the disaster’s long-term predictability.

Moreover, the fractional frequency and fractional difference estimation methodologies given in Sect. 3 shed light on the areas for future research. First of all, different functional forms could be used for the structural breaks. In this study, we showed that fractional frequency fits the structure of the Covid-19 epidemic quite well. However, another functional form can be recommended for another data type. Furthermore, different methodologies may be developed for implementing fractional difference estimation. Section 3.2 tried to examine the fractional differencing meaning and suggested an estimator that minimizes the information loss. The importance of taking differencing in different orders shows that new estimators and difference operators can be developed for various purposes in future studies.

## Data Availability

Data sharing not applicable to this article as publicly open datasets are available from the European Health Organization.

## Appendix A

This appendix provides asymptotic distribution of FFFFF-FI-ADF test statistics given in the text.

### Proof of Proposition 1

The proofs of (i) and (ii) are known (see Hamilton, 1994). By using the continuous mapping theorem, we can obtain the proofs of (iii) and (iv) as follows:

 □

### Proof of Theorem 1

Let $\sin (t) = \sin ( 2\pi k^{fr}t/T )$ and $\cos (t) = \cos ( 2\pi k^{fr}t/T )$. We first examine the demeaned case with $\beta = 0$ in Eq. (6). Let $y_{t}^{\mu }$ denote the OLS residuals from the demeaned case in the text with $w_{t} = ( 1,\sin (t),\cos (t) )'$

A.1 $$ y_{t}^{\mu } = \bar{\omega }_{t} - w_{t}' ( \hat{\theta } - \theta ) , $$

where $\theta = ( \alpha _{0},\alpha _{1},\alpha _{2} )^{\prime } $, *θ̂* is the OLS estimator of *θ*. We let $w = ( w_{1},\ldots,w_{T} )^{\prime } $, $\bar{\omega } = ( \bar{\omega }_{1},\ldots,\bar{\omega }_{T} )^{\prime } $ and $\mathbf{M}_{T} = \operatorname{diag} ( \sqrt{T},\sqrt{T},\sqrt{T} )$ to have

A.2 $$ \mathbf{M}_{T} ( \hat{\theta } - \theta ) = \bigl[ \mathbf{M}_{T}^{ - 1}w'w\mathbf{M}_{T}^{ - 1} \bigr]^{ - 1}\mathbf{M}_{T}^{ - 1}w'\bar{ \omega } . $$

Applying some algebra to (A.1) and (A.2),

A.3 $$ T^{ - 1/2}y_{ [ rT ]}^{\mu } = T^{ - 1/2}\bar{\omega }_{ [ rT ]}^{\mu } - T^{ - 1}w_{ [ rT ]}' \bigl[ \mathbf{M}_{T}^{ - 1}w'w \mathbf{M}_{T}^{ - 1} \bigr]^{ - 1} \mathbf{M}_{T}^{ - 1}w'\bar{\omega }. $$

Depending on FCLT, the first term of (A.3) becomes

A.4 $$ T^{ - 1/2}\bar{\omega }_{ [ rT ]} = T^{ - 1/2}\sum _{t = 1}^{ [ rT ]} \xi _{t} \to \sigma W(r). $$

The second component in (A.3) becomes

A.5 $$\begin{aligned} \begin{aligned} & \bigl[ \mathbf{M}_{T}^{ - 1}w'w \mathbf{M}_{T}^{ - 1} \bigr]^{ - 1} \\ &\quad = \begin{bmatrix} 1 & T^{ - 1}\sum_{t = 1}^{T} \sin (t) & T^{ - 1}\sum_{t = 1}^{T} \cos (t) \\ T^{ - 1}\sum_{t = 1}^{T} \sin (t) & T^{ - 1}\sum_{t = 1}^{T} \sin ^{2}(t) & T^{ - 1}\sum_{t = 1}^{T} \sin (t) T^{ - 1}\sum_{t = 1}^{T} \cos (t) \\ T^{ - 1}\sum_{t = 1}^{T} \cos (t) & T^{ - 1}\sum_{t = 1}^{T} \sin (t) T^{ - 1}\sum_{t = 1}^{T} \cos (t) & T^{ - 1}\sum_{t = 1}^{T} \cos ^{2}(t) \end{bmatrix}^{ - 1} \\ &\quad \to \begin{bmatrix} 1 & s_{0} & c_{0} \\ s_{0} & s_{2} & m_{0} \\ c_{0} & m_{0} & c_{2} \end{bmatrix}^{ - 1} = \frac{1}{\Delta _{1}} \begin{bmatrix} a_{11} & a_{12} & a_{13} \\ a_{12} & a_{22} & a_{23} \\ a_{13} & a_{23} & a_{33} \end{bmatrix} ,\end{aligned} \end{aligned}$$

where $\Delta _{1} = s_{2}c_{2} - s_{2}c_{0}^{2} - s_{0}^{2}c_{2} - m_{0}^{2}$, $a_{11} = s_{2}c_{2} - m_{0}^{2}$, $a_{12} = c_{0}m_{0} - s_{0}c_{2}$, $a_{13} = s_{0}m_{0} - s_{2}c_{0}$, $a_{22} = c_{2} - c_{0}^{2}$, $a_{23} = s_{0}c_{0} - m_{0}$ and $a_{33} = s_{2} - s_{0}^{2}$.

A.6 $$ T^{ - 1}\mathbf{M}_{T}^{ - 1}w'\bar{ \omega } = \begin{bmatrix} T^{ - 1/2}\sum_{t = 1}^{T} \bar{\omega }_{t} \\ T^{ - 1/2}\sum_{t = 1}^{T} \sin (t)\bar{\omega }_{t} \\ T^{ - 1/2}\sum_{t = 1}^{T} \cos (t)\bar{\omega }_{t} \end{bmatrix} \to \begin{bmatrix} \sigma f_{1} \\ \sigma f_{2} \\ \sigma f_{3} \end{bmatrix}. $$

Then

A.7 $$\begin{aligned} \begin{aligned} & T^{ - 1}w_{ [ rT ]}' \bigl[ \mathbf{M}_{T}^{ - 1}w'w \mathbf{M}_{T}^{ - 1} \bigr]^{ - 1} \mathbf{M}_{T}^{ - 1}w'\bar{\omega } \\ &\quad \to \frac{1}{\Delta _{1}} \begin{bmatrix} 1 & \sin ( 2\pi k^{fr}r ) & \cos ( 2\pi k^{fr}r ) \end{bmatrix} \begin{bmatrix} a_{11} & a_{12} & a_{13} \\ a_{21} & a_{22} & a_{23} \\ a_{31} & a_{32} & a_{33} \end{bmatrix} \begin{bmatrix} \sigma f_{1} \\ \sigma f_{3} \\ \sigma f_{4} \end{bmatrix} . \end{aligned} \end{aligned}$$

Finally, merging the outcomes in Eqs. (A.4) and (A.7), we obtain the demeaned Brownian motion by

A.8 $$ \begin{aligned} \frac{1}{\sigma \sqrt{T}} y_{ [ rT ]}^{\mu } &\to W_{\mu } \bigl( k^{fr},r \bigr) \\&= W ( r ) - \frac{\sigma }{\Delta _{1}} \begin{bmatrix} ( a_{11}f_{1} + a_{12}f_{3} + a_{13}f_{4} ) \\ {} + ( a_{21}f_{1} + a_{22}f_{3} + a_{23}f_{4} )\sin ( 2\pi k^{fr}r ) \\ {} + ( a_{31}f_{1} + a_{32}f_{3} + a_{33}f_{4} )\cos ( 2\pi k^{fr}r ) \end{bmatrix} . \end{aligned} $$

For the detrended case similar arguments follow so we skip the algebra. Using the above given results, under the null we can obtain the demeaned Brownian’s. Now we can proceed with the fractionally integrated part in the second step. Under the null hypothesis that $\bar{\omega }_{t}$ is a random walk and applying Lemma 1 and Lemma 2 and results in [18] and in [15], we obtain

$$ t_{FF,i} \bigl( k^{fr},d_{1} \bigr)\overset{w}{ \rightarrow}\frac{\smallint _{0}^{1}W_{i, - d_{1}} ( k^{fr},r )\,dB ( r )}{ ( \smallint _{0}^{1}W_{i, - d_{1}}^{2} ( k^{fr},r )\,dr )^{1/2}}, \quad i = \mu ,\tau \text{, if } 0 \le {d}_{1} < 0.5. $$

 □

## Appendix B: Critical values

**Table B1:** Critical values for FI models only intercept included in fractional frequency Fourier function $d = (0.1, 0.5)$

*T*	100			250			500		
	1%	5%	10%	1%	5%	10%	1%	5%	10%
*k* = 1.1									
0.1	−4.269	−3.601	−3.265	−4.140	−3.508	−3.180	−4.078	−3.481	−3.166
0.2	−3.981	−3.303	−2.982	−3.829	−3.243	−2.916	−3.876	−3.232	−2.906
0.3	−3.693	−3.043	−2.728	−3.618	−2.995	−2.641	−3.522	−2.949	−2.623
0.4	−3.523	−2.808	−2.457	−3.450	−2.768	−2.402	−3.326	−2.703	−2.355
0.5	−3.295	−2.610	−2.232	−3.166	−2.485	−2.118	−3.097	−2.449	−2.079
*k* = 1.2									
0.1	−4.166	−3.533	−3.179	−4.066	−3.442	−3.144	−4.103	−3.444	−3.108
0.2	−3.971	−3.259	−2.922	−3.816	−3.193	−2.852	−3.762	−3.188	−2.859
0.3	−3.650	−2.991	−2.675	−3.622	−2.970	−2.618	−3.644	−2.937	−2.601
0.4	−3.468	−2.774	−2.455	−3.337	−2.728	−2.373	−3.307	−2.663	−2.324
0.5	−3.292	−2.607	−2.211	−3.171	−2.486	−2.141	−3.105	−2.461	−2.071
*k* = 1.3									
0.1	−4.119	−3.429	−3.083	−4.024	−3.377	−3.047	−4.042	−3.377	−3.058
0.2	−3.988	−3.271	−2.935	−3.844	−3.200	−2.854	−3.744	−3.155	−2.833
0.3	−3.687	−3.021	−2.682	−3.593	−2.942	−2.625	−3.542	−2.931	−2.575
0.4	−3.516	−2.834	−2.475	−3.437	−2.705	−2.355	−3.290	−2.667	−2.315
0.5	−3.304	−2.584	−2.245	−3.188	−2.505	−2.148	−3.074	−2.472	−2.109
*k* = 1.4									
0.1	−4.091	−3.409	−3.046	−3.925	−3.352	−3.035	−3.942	−3.353	−3.004
0.2	−3.938	−3.234	−2.861	−3.805	−3.143	−2.782	−3.737	−3.151	−2.814
0.3	−3.625	−3.001	−2.649	−3.566	−2.936	−2.595	−3.529	−2.878	−2.561
0.4	−3.495	−2.760	−2.424	−3.409	−2.682	−2.371	−3.346	−2.677	−2.321
0.5	−3.302	−2.629	−2.263	−3.125	−2.474	−2.124	−3.117	−2.453	−2.091
*k* = 1.5									
0.1	−4.045	−3.355	−3.011	−3.939	−3.299	−2.936	−3.895	−3.288	−2.939
0.2	−3.850	−3.130	−2.782	−3.738	−3.079	−2.740	−3.714	−3.065	−2.746
0.3	−3.580	−2.941	−2.604	−3.476	−2.857	−2.516	−3.535	−2.876	−2.512
0.4	−3.420	−2.736	−2.392	−3.394	−2.715	−2.358	−3.263	−2.609	−2.276
0.5	−3.260	−2.550	−2.199	−3.126	−2.460	−2.103	−3.062	−2.422	−2.065
*k* = 1.6									
0.1	−3.991	−3.293	−2.923	−3.938	−3.254	−2.908	−3.929	−3.275	−2.920
0.2	−3.745	−3.105	−2.752	−3.711	−3.073	−2.737	−3.668	−3.065	−2.710
0.3	−3.619	−2.948	−2.573	−3.540	−2.857	−2.522	−3.440	−2.822	−2.482
0.4	−3.458	−2.740	−2.379	−3.337	−2.643	−2.293	−3.228	−2.608	−2.254
0.5	−3.208	−2.542	−2.169	−3.063	−2.445	−2.069	−3.079	−2.370	−2.030
*k* = 1.7									
0.1	−3.884	−3.261	−2.899	−3.922	−3.230	−2.864	−3.792	−3.208	−2.878
0.2	−3.748	−3.112	−2.733	−3.634	−3.019	−2.689	−3.644	−3.010	−2.645
0.3	−3.573	−2.886	−2.537	−3.499	−2.837	−2.491	−3.504	−2.809	−2.469
0.4	−3.448	−2.689	−2.340	−3.283	−2.635	−2.283	−3.269	−2.571	−2.213
0.5	−3.199	−2.510	−2.137	−3.102	−2.423	−2.072	−3.019	−2.397	−1.999
*k* = 1.8									
0.1	−3.870	−3.212	−2.856	−3.876	−3.194	−2.854	−3.817	−3.161	−2.825
0.2	−3.727	−3.058	−2.693	−3.672	−3.023	−2.669	−3.592	−2.992	−2.648
0.3	−3.524	−2.846	−2.497	−3.488	−2.845	−2.493	−3.406	−2.749	−2.413
0.4	−3.319	−2.685	−2.330	−3.294	−2.624	−2.261	−3.241	−2.589	−2.236
0.5	−3.225	−2.520	−2.149	−3.015	−2.398	−2.055	−3.017	−2.361	−2.008
*k* = 1.9									
0.1	−3.894	−3.198	−2.814	−3.805	−3.164	−2.824	−3.771	−3.156	−2.807
0.2	−3.712	−3.016	−2.650	−3.642	−3.003	−2.651	−3.623	−2.962	−2.626
0.3	−3.480	−2.830	−2.484	−3.445	−2.818	−2.448	−3.458	−2.773	−2.415
0.4	−3.347	−2.678	−2.296	−3.178	−2.567	−2.231	−3.223	−2.540	−2.185
0.5	−3.179	−2.477	−2.122	−3.057	−2.415	−2.072	−3.002	−2.372	−2.016
*k* = 2.0									
0.1	−3.852	−3.165	−2.797	−3.837	−3.119	−2.777	−3.737	−3.111	−2.770
0.2	−3.652	−3.030	−2.655	−3.604	−2.963	−2.634	−3.537	−2.930	−2.594
0.3	−3.533	−2.856	−2.472	−3.480	−2.782	−2.409	−3.361	−2.742	−2.414
0.4	−3.265	−2.636	−2.272	−3.263	−2.590	−2.221	−3.186	−2.556	−2.212
0.5	−3.215	−2.500	−2.143	−3.053	−2.379	−2.010	−2.941	−2.312	−1.965

**Table B2:** Critical values for FI models only intercept included in fractional frequency Fourier function $d = (0.6, 0.9)$

*T*	100			250			500		
	1%	5%	10%	1%	5%	10%	1%	5%	10%
*k* = 1.1									
0.6	−2.994	−2.367	−2.017	−2.925	−2.297	−1.915	−2.851	−2.182	−1.828
0.7	−2.836	−2.180	−1.829	−2.718	−2.057	−1.724	−2.667	−2.021	−1.656
0.8	−2.743	−2.074	−1.709	−2.649	−1.926	−1.575	−2.551	−1.877	−1.513
0.9	−2.643	−1.963	−1.579	−2.549	−1.824	−1.461	−2.512	−1.832	−1.436
*k* = 1.2									
0.6	−3.055	−2.375	−2.027	−2.962	−2.279	−1.927	−2.819	−2.194	−1.843
0.7	−2.917	−2.172	−1.838	−2.787	−2.113	−1.723	−2.709	−2.035	−1.662
0.8	−2.847	−2.062	−1.704	−2.657	−1.988	−1.636	−2.575	−1.891	−1.520
0.9	−2.597	−1.920	−1.557	−2.545	−1.852	−1.480	−2.400	−1.796	−1.441
*k* = 1.3									
0.6	−3.161	−2.431	−2.063	−2.962	−2.282	−1.928	−2.880	−2.208	−1.856
0.7	−2.909	−2.225	−1.845	−2.797	−2.114	−1.780	−2.712	−2.043	−1.690
0.8	−2.859	−2.131	−1.763	−2.704	−1.962	−1.611	−2.614	−1.908	−1.518
0.9	−2.678	−1.985	−1.610	−2.582	−1.880	−1.500	−2.465	−1.813	−1.449
*k* = 1.4									
0.6	−3.074	−2.425	−2.075	−2.893	−2.260	−1.915	−2.837	−2.221	−1.866
0.7	−2.903	−2.190	−1.846	−2.816	−2.085	−1.744	−2.723	−2.079	−1.711
0.8	−2.786	−2.094	−1.742	−2.652	−1.984	−1.641	−2.676	−1.899	−1.545
0.9	−2.715	−1.984	−1.633	−2.623	−1.887	−1.527	−2.436	−1.777	−1.449
*k* = 1.5									
0.6	−3.031	−2.417	−2.036	−2.865	−2.229	−1.901	−2.946	−2.181	−1.824
0.7	−2.875	−2.231	−1.883	−2.750	−2.116	−1.763	−2.765	−2.045	−1.692
0.8	−2.840	−2.113	−1.746	−2.724	−1.982	−1.615	−2.570	−1.917	−1.575
0.9	−2.699	−1.954	−1.608	−2.528	−1.868	−1.488	−2.469	−1.804	−1.456
*k* = 1.6									
0.6	−3.078	−2.377	−2.016	−2.914	−2.247	−1.898	−2.875	−2.168	−1.840
0.7	−2.942	−2.219	−1.825	−2.755	−2.105	−1.746	−2.666	−1.990	−1.650
0.8	−2.760	−2.081	−1.718	−2.646	−2.010	−1.604	−2.580	−1.901	−1.537
0.9	−2.619	−1.974	−1.598	−2.497	−1.812	−1.468	−2.519	−1.797	−1.422
*k* = 1.7									
0.6	−3.076	−2.382	−1.997	−2.923	−2.203	−1.863	−2.871	−2.215	−1.829
0.7	−2.898	−2.194	−1.828	−2.755	−2.086	−1.723	−2.654	−1.979	−1.633
0.8	−2.740	−2.064	−1.707	−2.712	−1.980	−1.620	−2.604	−1.899	−1.539
0.9	−2.698	−1.983	−1.614	−2.565	−1.846	−1.528	−2.470	−1.786	−1.431
*k* = 1.8									
0.6	−3.054	−2.339	−1.988	−2.964	−2.237	−1.867	−2.837	−2.154	−1.796
0.7	−2.890	−2.185	−1.807	−2.781	−2.078	−1.715	−2.687	−2.004	−1.636
0.8	−2.828	−2.084	−1.697	−2.652	−1.990	−1.606	−2.532	−1.880	−1.503
0.9	−2.744	−1.976	−1.598	−2.545	−1.880	−1.511	−2.466	−1.811	−1.460
*k* = 1.9									
0.6	−3.038	−2.300	−1.946	−2.897	−2.229	−1.865	−2.865	−2.165	−1.785
0.7	−2.832	−2.186	−1.808	−2.761	−2.109	−1.713	−2.602	−1.983	−1.645
0.8	−2.726	−2.028	−1.667	−2.595	−1.964	−1.591	−2.577	−1.872	−1.495
0.9	−2.634	−1.901	−1.568	−2.497	−1.852	−1.496	−2.477	−1.789	−1.446

**Table B3:** Critical values for FI models intercept and trend included in fractional frequency Fourier function $d = (0.1, 0.5)$

*T*	100			250			500		
	1%	5%	10%	1%	5%	10%	1%	5%	10%
*k* = 1.1									
0.1	−4.813	−4.163	−3.830	−4.667	−4.078	−3.772	−4.587	−4.012	−3.733
0.2	−4.474	−3.866	−3.557	−4.353	−3.772	−3.436	−4.343	−3.728	−3.443
0.3	−4.261	−3.566	−3.234	−4.060	−3.434	−3.106	−3.958	−3.390	−3.067
0.4	−3.904	−3.253	−2.909	−3.718	−3.116	−2.783	−3.673	−3.035	−2.715
0.5	−3.656	−2.988	−2.636	−3.488	−2.827	−2.468	−3.376	−2.734	−2.411
*k* = 1.2									
0.1	−4.737	−4.146	−3.811	−4.622	−4.018	−3.730	−4.530	−4.012	−3.729
0.2	−4.497	−3.853	−3.533	−4.367	−3.753	−3.429	−4.252	−3.712	−3.409
0.3	−4.185	−3.524	−3.200	−4.012	−3.407	−3.105	−3.954	−3.339	−3.036
0.4	−3.918	−3.251	−2.906	−3.706	−3.108	−2.786	−3.623	−3.037	−2.695
0.5	−3.639	−2.997	−2.616	−3.515	−2.811	−2.483	−3.350	−2.759	−2.393
*k* = 1.3									
0.1	−4.720	−4.141	−3.821	−4.625	−4.072	−3.755	−4.565	−3.982	−3.708
0.2	−4.463	−3.859	−3.538	−4.316	−3.765	−3.455	−4.263	−3.686	−3.384
0.3	−4.168	−3.548	−3.213	−4.023	−3.417	−3.084	−3.993	−3.366	−3.032
0.4	−3.874	−3.223	−2.905	−3.695	−3.061	−2.750	−3.630	−3.036	−2.698
0.5	−3.610	−2.962	−2.615	−3.437	−2.815	−2.462	−3.310	−2.735	−2.404
*k* = 1.4									
0.1	−4.720	−4.134	−3.814	−4.685	−4.012	−3.711	−4.570	−3.992	−3.683
0.2	−4.364	−3.785	−3.467	−4.258	−3.678	−3.375	−4.222	−3.669	−3.379
0.3	−4.172	−3.514	−3.189	−3.957	−3.373	−3.062	−3.906	−3.320	−2.992
0.4	−3.931	−3.228	−2.890	−3.685	−3.055	−2.717	−3.651	−3.015	−2.681
0.5	−3.618	−2.957	−2.591	−3.410	−2.781	−2.463	−3.298	−2.674	−2.375
*k* = 1.5									
0.1	−4.638	−4.086	−3.750	−4.552	−3.986	−3.675	−4.509	−3.950	−3.631
0.2	−4.389	−3.769	−3.442	−4.223	−3.676	−3.348	−4.213	−3.654	−3.338
0.3	−4.116	−3.485	−3.161	−4.018	−3.364	−3.022	−3.906	−3.320	−3.001
0.4	−3.843	−3.206	−2.852	−3.704	−3.044	−2.726	−3.665	−3.036	−2.680
0.5	−3.626	−2.949	−2.587	−3.386	−2.764	−2.429	−3.309	−2.652	−2.307
*k* = 1.6									
0.1	−4.739	−4.055	−3.749	−4.557	−3.965	−3.672	−4.476	−3.928	−3.624
0.2	−4.422	−3.736	−3.423	−4.207	−3.638	−3.312	−4.195	−3.599	−3.292
0.3	−4.157	−3.477	−3.134	−3.954	−3.361	−3.032	−3.893	−3.292	−2.954
0.4	−3.885	−3.147	−2.808	−3.710	−3.045	−2.699	−3.571	−2.973	−2.640
0.5	−3.594	−2.897	−2.559	−3.380	−2.764	−2.405	−3.367	−2.692	−2.335
*k* = 1.7									
0.1	−4.651	−3.990	−3.653	−4.493	−3.905	−3.584	−4.447	−3.854	−3.560
0.2	−4.374	−3.776	−3.401	−4.323	−3.642	−3.313	−4.249	−3.557	−3.264
0.3	−4.078	−3.428	−3.088	−3.940	−3.319	−3.000	−3.858	−3.269	−2.975
0.4	−3.800	−3.159	−2.815	−3.694	−3.064	−2.687	−3.583	−2.987	−2.630
0.5	−3.480	−2.861	−2.523	−3.419	−2.754	−2.398	−3.297	−2.660	−2.317
*k* = 1.8									
0.1	−4.580	−3.944	−3.633	−4.484	−3.890	−3.568	−4.469	−3.877	−3.555
0.2	−4.327	−3.710	−3.372	−4.225	−3.582	−3.296	−4.153	−3.561	−3.253
0.3	−4.037	−3.411	−3.068	−3.922	−3.279	−2.971	−3.846	−3.247	−2.929
0.4	−3.756	−3.161	−2.814	−3.643	−3.013	−2.670	−3.628	−2.955	−2.631
0.5	−3.595	−2.892	−2.547	−3.440	−2.754	−2.399	−3.326	−2.659	−2.315
*k* = 1.9									
0.1	−4.644	−3.926	−3.587	−4.448	−3.811	−3.471	−4.367	−3.796	−3.490
0.2	−4.263	−3.670	−3.333	−4.071	−3.506	−3.226	−4.167	−3.520	−3.203
0.3	−4.079	−3.395	−3.067	−3.898	−3.275	−2.934	−3.838	−3.234	−2.900
0.4	−3.793	−3.105	−2.769	−3.663	−3.028	−2.679	−3.535	−2.916	−2.586
0.5	−3.635	−2.871	−2.517	−3.402	−2.741	−2.415	−3.305	−2.648	−2.308
*k* = 2.0									
0.1	−4.537	−3.880	−3.531	−4.398	−3.815	−3.458	−4.318	−3.719	−3.429
0.2	−4.257	−3.632	−3.283	−4.210	−3.568	−3.235	−4.128	−3.463	−3.157
0.3	−4.055	−3.368	−3.003	−3.869	−3.261	−2.936	−3.776	−3.171	−2.841
0.4	−3.757	−3.086	−2.750	−3.573	−2.977	−2.652	−3.575	−2.914	−2.583
0.5	−3.512	−2.875	−2.499	−3.326	−2.706	−2.354	−3.259	−2.638	−2.313

**Table B4:** Critical values for FI models intercept and trend included in fractional frequency Fourier function $d = (0.6, 0.9)$

*T*	100			250			500		
	1%	5%	10%	1%	5%	10%	1%	5%	10%
*k* = 1.1									
0.6	−3.405	−2.730	−2.373	−3.221	−2.558	−2.197	−3.076	−2.452	−2.103
0.7	−3.124	−2.496	−2.147	−2.979	−2.341	−1.977	−2.900	−2.184	−1.847
0.8	−3.037	−2.319	−1.944	−2.838	−2.163	−1.797	−2.738	−2.020	−1.665
0.9	−2.870	−2.164	−1.797	−2.722	−2.005	−1.614	−2.603	−1.897	−1.550
*k* = 1.2									
0.6	−3.373	−2.730	−2.361	−3.215	−2.558	−2.209	−3.132	−2.475	−2.121
0.7	−3.168	−2.528	−2.164	−3.048	−2.320	−1.968	−2.950	−2.201	−1.852
0.8	−2.974	−2.318	−1.975	−2.859	−2.133	−1.789	−2.739	−2.075	−1.717
0.9	−2.927	−2.140	−1.759	−2.619	−2.018	−1.648	−2.523	−1.905	−1.565
*k* = 1.3									
0.6	−3.346	−2.675	−2.342	−3.202	−2.545	−2.203	−3.028	−2.441	−2.083
0.7	−3.195	−2.487	−2.132	−3.042	−2.324	−1.957	−2.949	−2.243	−1.862
0.8	−3.027	−2.300	−1.937	−2.855	−2.139	−1.768	−2.659	−1.995	−1.650
0.9	−2.862	−2.147	−1.771	−2.702	−1.976	−1.604	−2.593	−1.880	−1.523
*k* = 1.4									
0.6	−3.391	−2.717	−2.350	−3.200	−2.521	−2.167	−3.046	−2.440	−2.090
0.7	−3.167	−2.476	−2.103	−3.006	−2.321	−1.982	−2.909	−2.214	−1.852
0.8	−3.002	−2.283	−1.935	−2.780	−2.113	−1.769	−2.714	−2.013	−1.643
0.9	−2.802	−2.139	−1.773	−2.708	−1.978	−1.603	−2.637	−1.904	−1.537
*k* = 1.5									
0.6	−3.401	−2.734	−2.357	−3.176	−2.514	−2.151	−3.038	−2.382	−2.037
0.7	−3.098	−2.461	−2.072	−2.970	−2.308	−1.922	−2.839	−2.155	−1.802
0.8	−2.982	−2.284	−1.917	−2.759	−2.105	−1.749	−2.665	−2.025	−1.665
0.9	−2.902	−2.172	−1.752	−2.667	−2.010	−1.623	−2.517	−1.889	−1.501
*k* = 1.6									
0.6	−3.363	−2.667	−2.330	−3.117	−2.457	−2.111	−3.068	−2.415	−2.044
0.7	−3.137	−2.435	−2.089	−2.904	−2.245	−1.902	−2.829	−2.163	−1.813
0.8	−3.035	−2.268	−1.917	−2.801	−2.135	−1.772	−2.707	−2.023	−1.662
0.9	−2.827	−2.118	−1.752	−2.655	−1.945	−1.590	−2.577	−1.913	−1.542
*k* = 1.7									
0.6	−3.350	−2.663	−2.302	−3.242	−2.531	−2.163	−3.118	−2.461	−2.069
0.7	−3.157	−2.442	−2.075	−2.988	−2.308	−1.944	−2.800	−2.164	−1.823
0.8	−2.985	−2.287	−1.917	−2.760	−2.095	−1.742	−2.651	−2.013	−1.653
0.9	−2.889	−2.142	−1.739	−2.601	−1.952	−1.597	−2.595	−1.878	−1.501
*k* = 1.8									
0.6	−3.295	−2.623	−2.268	−3.177	−2.474	−2.145	−3.055	−2.394	−2.032
0.7	−3.102	−2.451	−2.105	−2.926	−2.250	−1.912	−2.866	−2.212	−1.824
0.8	−3.015	−2.289	−1.909	−2.793	−2.125	−1.745	−2.686	−1.965	−1.629
0.9	−2.831	−2.144	−1.756	−2.698	−1.973	−1.597	−2.628	−1.895	−1.537
*k* = 1.9									
0.6	−3.273	−2.653	−2.293	−3.162	−2.497	−2.137	−3.096	−2.377	−2.019
0.7	−3.192	−2.473	−2.101	−3.014	−2.300	−1.942	−2.890	−2.196	−1.826
0.8	−2.893	−2.265	−1.907	−2.790	−2.103	−1.754	−2.694	−2.021	−1.673
0.9	−2.792	−2.127	−1.767	−2.633	−1.943	−1.582	−2.514	−1.853	−1.514

## Footnotes

1 See [10] for more details.

2 $\Gamma (\cdot )$ denotes the gamma function, and $\{\pi _{i}(d)\}$ represents the sequence of coefficients associated with the expansion of $\Delta ^{d}$ in powers of *L*, which are defined as $\pi _{i}(d) = \frac{\Gamma (i - d)}{\Gamma ( - d)\Gamma (i - 1)}$.

3 The results can be provided upon request.

4 Since the research of these topics is beyond the scope of the study, the subject is left at this maturity level. However, more detailed discussions on these issues are considered for future studies.

5 The country codes of these countries are given in the Appendix.
